# From stretch﻿able materials and functional fibers to intelligent textile sensing systems

**DOI:** 10.1038/s43246-026-01333-4

**Published:** 2026-10-21

**Authors:** Maria Papaefstathiou, Mohamed Elgendi, Carlo Menon

**Affiliations:** 1 Biomedical and Mobile Health Technology (BMHT) Lab, Department of Health Sciences and Technology, ETH Zurich, Zurich, Switzerland; 2 Department of Biomedical Engineering and Biotechnology, Khalifa University of Science and Technology, Abu Dhabi, UAE; 3 Healthcare Engineering Innovation Group (HEIG), Khalifa University of Science and Technology, Abu Dhabi, UAE

**Keywords:** Quality of life, Biomedical engineering, Polymers, Sensors and biosensors

## Abstract

Wearable sensors that offer significant adaptability and comfort demonstrate substantial potential for discrete, seamless integration into garments, enabling diverse applications for remote monitoring. The transformation of rigid electronic devices into flexible and stretchable sensors, achieved by integrating conductive materials with compliant dielectrics, has enabled the development of robust sensors. However, several factors must be addressed to enable widespread adoption, including garment integration, wireless communication, data pipelines, signal processing, multimodal fusion, privacy and security concerns. Addressing these challenges is essential for establishing the technological and social infrastructure necessary for the widespread adoption of wearable technologies. This review highlights innovations in material and mechanical design strategies that have shaped the current landscape of wearables, and points out some remaining challenges.

## Abstract (web summary)

Stretchable wearable sensors enable comfortable garment integration for remote monitoring, but advances in connectivity, data processing, privacy, and security are needed for widespread adoption. Here, the authors highlight innovations in materials and mechanical design and identify key challenges for wearable technologies.

## Introduction

Traditional sensors and motion-monitoring devices have typically relied on rigid electronic components, such as silicon-based electronics, limiting their flexibility and suitability for long-term wearable applications. Moreover, these devices were prone to mechanical failure due to repeated bending and stretching induced by body movements^1^. Their rigidity also leads to a less comfortable experience, especially during prolonged wear. To address these challenges, soft materials that exhibit high sensitivity and flexibility have emerged as a solution^2^. Soft, stretchable materials require flexibility to adapt and maintain conformal contact during dynamic movement, allowing high-fidelity bio-signal acquisition^3–5^. Over the past few years, substantial effort has been devoted to the development of flexible elastomeric systems that incorporate conductive fillers that provide high electrical conductivity, cyclic stability, and reduced signal noise during dynamic deformation^6–11^. More specifically, recent specialized reviews explored wearable textile electronics from multiple perspectives, including mitigation of motion artifacts in soft bioelectronics^6^ and material-based approaches to improve sensor sensitivity^7^. In parallel, several review papers have summarized advances in fiber electronics, their constituent materials and applications^8^, as well as textile fabrication techniques and hierarchical fiber-yarn-fabric structures^9^. Subsequent work has expanded this perspective toward textile-integrated healthcare systems, encompassing physiological and biochemical sensing, therapeutic garments, textile body-area networks, energy-autonomous operation, embedded computing, data governance, regulation, and clinical translation^10^, while other work on System-on-Fiber (SoF) technologies has enabled the integration of sensing, computation, memory, communication and neuromorphic functions directly within fiber architectures^11^.

Although numerous specialized reviews have summarized advances in sensing modalities, there is little attention to the systematic evaluation of trade-offs between sensing characteristics and practical implementation requirements, including sensitivity, stretchability, durability and fabrication complexity. This Review addresses this gap by providing a comparative assessment of the trade-offs among sensing performance, mechanical requirements, fabrication considerations, and textile integration, while identifying the strengths and limitations of current design approaches. Moreover, there is limited cross-disciplinary synthesis. Most reviews address individual sub-fields such as materials, conductive circuits, wireless communications and data processing separately, with little focus on their intersections. Consequently, cross-disciplinary knowledge is often overlooked. A related gap is that limited attention has also been paid to manufacturing scalability, despite its importance for translating wearable technologies from laboratory-scale demonstrations to reliable, large-scale production. In addition, ethical considerations and data-security constraints represent important limitations that must be addressed to enable responsible large-scale deployment. These concerns become particularly significant as wearable technologies are  integrated into emerging social infrastructures and artificial intelligence (AI)-driven systems where they introduce new challenges related to privacy, data governance, and ethical use.  Unresolved ethical and data-security challenges highlight the need for appropriate social and regulatory frameworks that protect data and prioritize human rights and safety to ensure responsible widespread adoption. As wearable technologies increasingly evolve into multifunctional integrated platforms, a broader review that connects material preparation, fabrication methods, sensing strategies, instrumentation, wireless communication, signal processing, and system-level integration remains highly valuable.

**Fig. 1:**
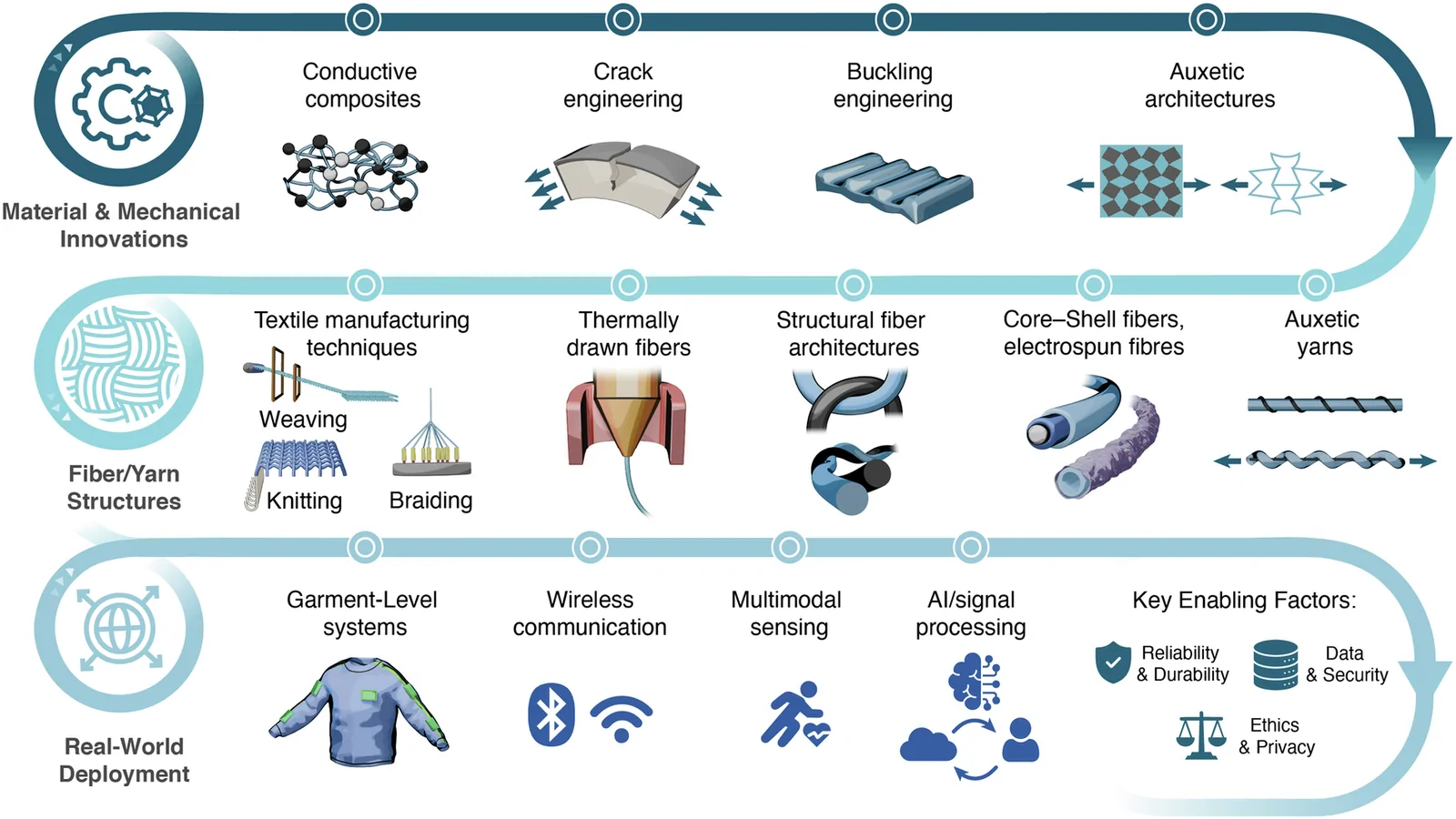
Overview and roadmap of the review. The field has progressed from developing functional materials such as conductive composites and sensitivity-enhancing designs (e.g., crack engineering, buckling engineering, auxetic metamaterials) to advanced fiber and yarn-based architectures, such as auxetic yarns, interlocking structures, and textile-integrated garment systems, with simultaneous focus on manufacturability, fabrication methods, wireless communication, multimodal sensing, and the integration of artificial intelligence (AI) and signal processing. However, challenges, including reliability, durability, data security, privacy, and ethical concerns, must be addressed to support widespread adoption.

In this Review, we discuss the latest strategies in materials science and structural engineering, as well as those used to optimize sensitivity and stretchability, the device/platform evolution from thin-films to fiber/yarn-based electronics, fabrication methods that facilitated the scalable manufacturability, and garment-level system integration for the development of large-area sensors and conformable systems. An overview of the topics covered in this review is provided in Fig. 1. Practical applications are also discussed to highlight their impact on real-world deployment. Finally, we explore key challenges, including reliability, washability, data security, privacy, and ethical considerations, that must be addressed to enable the next generation of intelligent closed-loop wearable systems for personalized healthcare.

## Material and mechanical innovations enabling the transition from rigid to stretchable wearable systems

To overcome the limitations of rigid electronic devices, researchers have implemented various structural designs to improve the stretchability of flexible electronics^12,13^. The progress of intrinsically soft electronics depends on the development of materials that exhibit high electrical conductivity and exceptional deformability^14,15^. Metallic nanomaterials, such as zero-dimensional (0D) nanoparticles, one-dimensional (1D) nanowires (NWs) and two-dimensional (2D) materials (such as graphene), are essential for the fabrication of conductive and stretchable composites. Embedding these nanomaterials into elastomeric or other soft matrices enables the formation of conductive networks that retain electrical performance under large mechanical deformations. Some of the most commonly used polymeric materials for a wide range of strains are thermoplastic polyurethane (TPU)^16^, Ecoflex^17^, and poly(dimethylsiloxane) (PDMS)^18^, which offer superior stretchability and flexibility to the sensor. Therefore, the realization of wearable strain sensors based on polymer/filler composites with outstanding performance, including superior stretchability and a wide sensing range, requires consideration of several aspects summarized in Box 1.

**Fig. 2:**
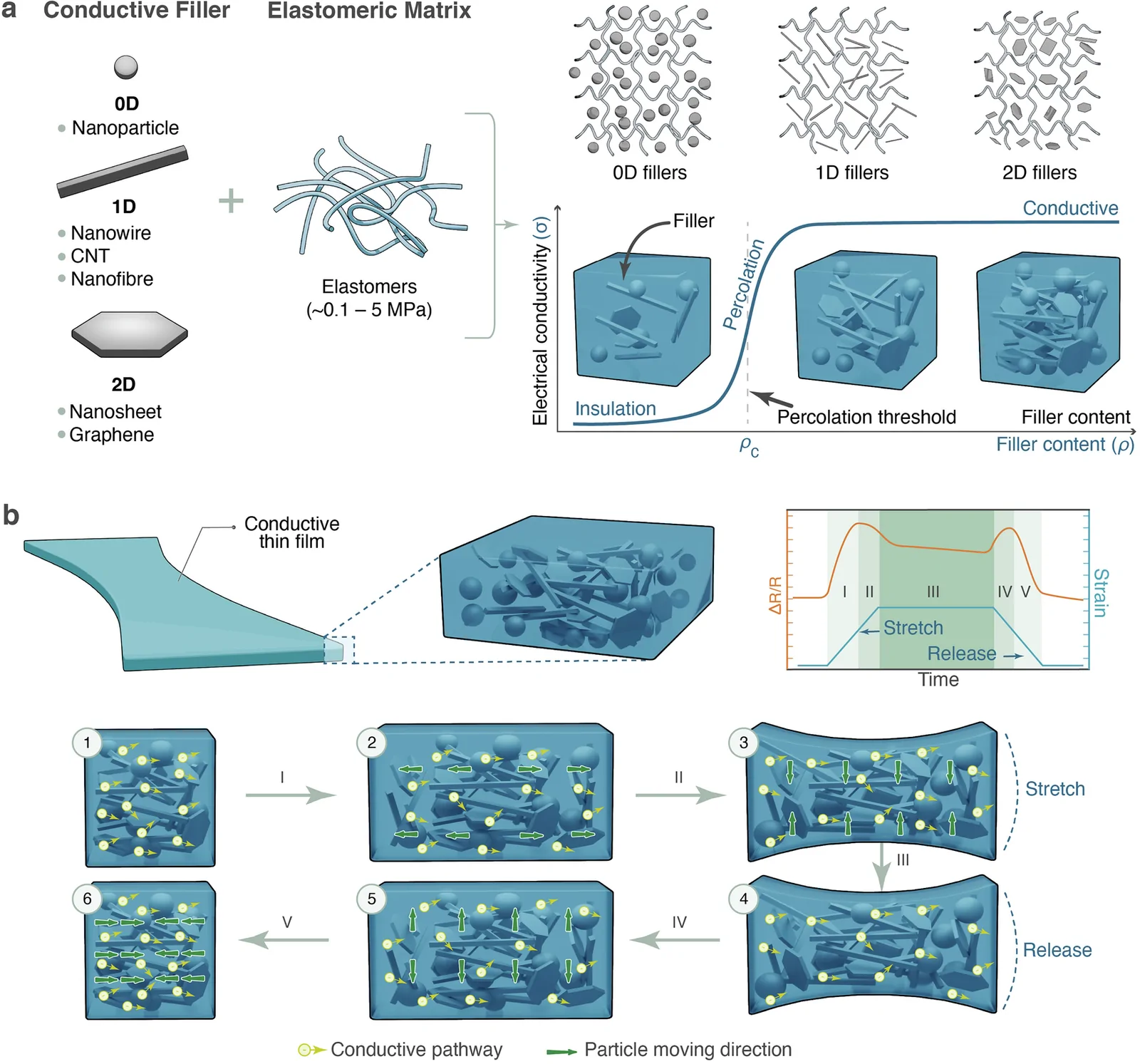
Conductive filler networks, percolation behavior, and piezoresistive response during mechanical deformation. **a** Schematic illustration of conductive fillers with different dimensionalities (0D, 1D, and 2D) embedded within elastomeric matrices, together with the percolation threshold, defined as the critical filler concentration at which a continuous conductive network forms throughout the elastomer matrix. Adapted from ref. ^333, Copyright © 2022, with permission from Elsevier, and ref. 334^, under the terms of the CC BY 4.0 license. © 2024 The Authors. **b** Schematic illustration of the piezoresistive response of a conductive thin film during a stretch–release cycle. Applied strain induces rearrangement of the conductive components and alters the conductive pathways within the film. Upon release, the conductive network progressively recovers toward its initial configuration. Yellow symbols indicate conductive pathways, and green arrows indicate the direction of particle movement. Adapted from ref. ^332^, © 2022 The Authors. Advanced Functional Materials published by Wiley-VCH GmbH.

Box 1 Operational mechanisms of piezoresistive strain sensors An important factor to consider in developing conductive polymers is the percolation network theory and the critical filler concentration, known as the percolation threshold^311^. Conductivity in stretchable composites arises from dynamic percolation networks formed by nanoscale fillers within elastomeric matrices. Percolation theory provides a quantitative and conceptual framework for understanding how filler morphology, including particle shape, dimensionality, aspect ratio and distribution govern the onset and stability of electrical pathways^312^. Specifically, there is a critical filler concentration, known as the percolation threshold (*ρ*_*c*_). Below *ρ*_*c*_, no continuous conductive pathway spans the material. At *ρ*_*c*_, a critical conductive network emerges, whereas above *ρ*_*c*_, continuous conductive pathways are established where the network percolates (Fig. 2a). For conductive composites with filler concentrations above the percolation threshold, electrical transport can be modeled as a network of resistive pathways, with the resistance strongly dependent on the distance between neighboring conductive nanoparticles in the insulating matrix^313–315^. Therefore, the transition from an insulator to a conductor upon adding conductive fillers can be calculated by Equation (1).

1 $$\sigma ={\sigma }_{0}{(\rho -{\rho }_{c})}^{t}\quad \,{\mbox{for}}\,\rho > {\rho }_{c}$$

In the low-concentration region, the electrical conductivity (*σ*) increases nonlinearly with a slight increase in the concentration of the conductive filler up to a certain concentration. The overall resistance is influenced by several parameters, including the polymer’s dielectric properties, the tunneling gap between adjacent conductive fillers, and the fillers’ electronic structure^316^. At percolation, conductive paths begin to build, allowing the current to pass through the conductive fillers, which come into contact with each other^97,317^. These factors are mainly the dispersion preparation process, the aspect ratio of the fillers, the orientation, state, and polymer viscosity, among others^318–321^. The gauge factor (*GF*), a measure of piezoresistive sensitivity, can be calculated using Equation (2).

2 $$GF=\frac{\Delta R/{R}_{0}}{\varepsilon }$$

As illustrated by the numerical states in Fig. 2b, the piezoresistive response originates from strain-induced changes in the conductive network. In the initial state (State 1), the conductive fillers form electrical pathways through direct contacts and/or electron tunneling across small interparticle gaps. During the initial stretching stage (State 1 to State 2, Process I), deformation of the elastomeric matrix causes the conductive fillers to move and the conductive network to rearrange. With further stretching (State 2 to State 3, Process II), the interparticle distances increase, and some conductive contacts are disrupted, thereby modifying the number and configuration of conductive pathways. The increased separation between neighboring conductive fillers also alters electron tunneling, whose probability is strongly dependent on the tunneling distance. These strain-induced changes in the conductive network produce a measurable change in electrical resistance, converting mechanical deformation into an electrical signal. Upon release of the applied strain (State 3 to State 4, Process III), the elastomeric matrix begins to recover, and the conductive fillers move toward their original relative positions. As recovery proceeds (State 4 to State 5, Process IV), conductive contacts and tunneling pathways are progressively restored, and the resistance approaches its initial value. During the final recovery stage (State 5 to State 6, Process V), the conductive network approaches its initial configuration, allowing the sensor to return toward its baseline electrical state.

### Highly stretchable composites

For example, both graphene nanopapers^19^ and super-aligned carbon nanotube (SACNT) films^20^ have been engineered to maintain electrical integrity under large strains, achieving stretchability beyond that of traditional thin-film sensors. Together, these studies^19,20^ demonstrate the potential of hierarchical conductive architectures to achieve high stretchability (up to 100% and 400%, respectively). Therefore, a uniform dispersion significantly improves the conductive pathways of conductive fillers within the polymer matrix. Appropriate mixing parameters, such as time, temperature, compatibility, and concentration, are therefore crucial for optimization^21–24^. Non-uniform dispersion can hinder effective charge transport and introduce localized stress concentrations, potentially leading to cracking during film formation. Maintaining an appropriate balance between the metallic and polymeric components is essential for mechanical reliability, as compositional deviations or poor processability often lead to cracking.

In a different study, drawing inspiration from skin tissue, the design incorporates nanofibers to disperse stress and wrinkle structures to enhance stretchability and durability. This design resulted in the development of nanofiber-reinforced elastic conductors with excellent mechanical durability (excellent performance maintained after 5000 cycles at 50% strain) and conductivity^25^. More specifically, the composite was fabricated by stencil printing onto electrospun poly(vinylidene fluoride) (PVDF) nanofibers, which exhibit strong adhesion to the matrix. The resulting nanofiber-reinforced composite, containing randomly aligned polymer nanofibers with 75% metal fillers embedded in a silver-fluoroelastomer matrix, was 325 times tougher and more resistant to crack propagation. Although excessive filler loading often results in increased sheet resistance, which in turn leads to weak electrical signals, poor signal-to-noise ratios, higher power requirements, and non-uniform current distribution. If the film is too conductive, the baseline resistance is very small, and strain-induced changes become difficult to detect, therefore, highly stretchable composites are designed to sustain large deformations while maintaining their functional properties.

**Fig. 3:**
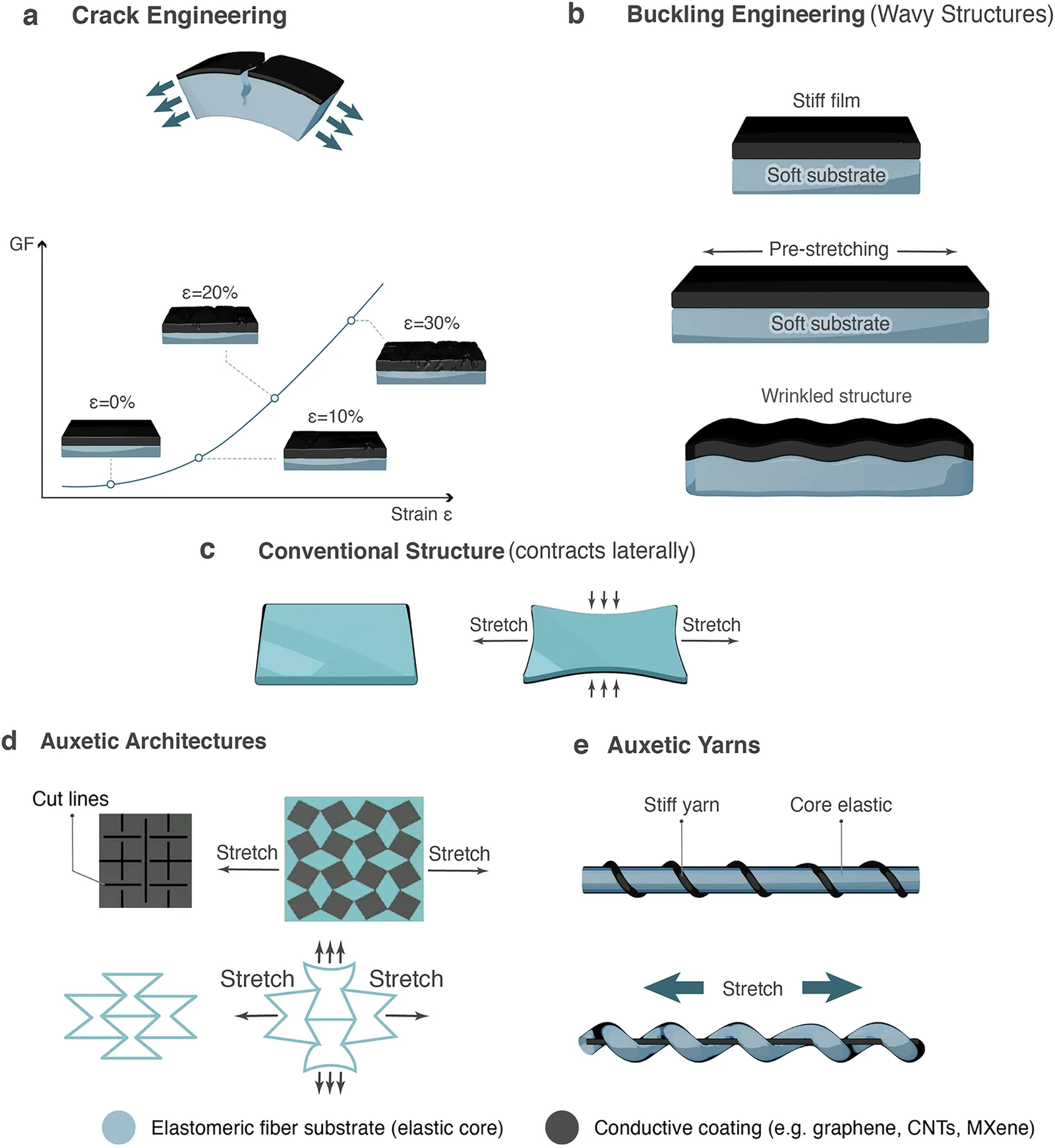
Schematic representation of some representative deformation mechanisms of different stretchable structural designs. **a** Crack engineering: The formation of microcracks through prestretching enables strain accommodation and significantly enhances sensitivity, resulting in high gauge factors (*GF*). Adapted from ref. ^34^, with permission from John Wiley & Sons. **b** Buckling engineering: Prestretching of elastomeric substrates induces wavy or buckled structures upon release, enabling large deformations while maintaining electrical and mechanical integrity. Adapted from refs. ^42^, with permission from John Wiley & Sons. **c** Conventional structures typically exhibit a positive Poisson’s ratio, resulting in lateral contraction when stretched. **d** Auxetic architectures: A wide range of geometries, such as rectangular patterns and re-entrant honeycombs, have been developed to achieve negative Poisson’s ratio behavior and enhanced deformability. Adapted from refs. ^47^ under the terms of the CC BY 4.0 license, and from ref. ^51^under the terms of the CC BY-NC 3.0 license. © 2017 The Author(s). Published by the Royal Society of Chemistry. **e** Auxetic yarns: Specialized yarn geometries enable negative Poisson’s ratio behavior, resulting in lateral expansion upon stretching and enhanced functionality for wearable applications^52^. Adapted from ref. ^52^ © 2023 The Authors. Advanced Materials published by Wiley-VCH GmbH.

Hence,  an important factor when a high volume of conductive fillers is introduced to the much softer elastomeric matrices is that fillers can act as defects, initiating crack growth within the elastomeric matrix and compromising the sensor’s structural integrity over time. Importantly, such crack formation can also contribute to enhanced sensing sensitivity, as the current/resistance is exponentially dependent on the distance between cracks^26^. Consequently, even a minuscule deformation of the cracks can result in a large, measurable change in the electrical signal (Fig. 3a)^26^. This leads to very high gauge factors compared to traditional strain gauges, which typically rely on a linear change in resistance due to simple geometric changes^27^. Therefore, the controlled formation and propagation of microcracks can generate highly strain-sensitive conductive pathways, resulting in ultrahigh gauge factors and enabling the detection of minute physiological signals. Consequently, optimizing the intrinsic material properties can improve the performance of piezoresistive sensors, with controlled crack formation serving as an effective strategy for enhancing strain-induced resistance changes^26^. As a result, crack-engineered strategies have attracted considerable attention for their ability to amplify resistance variations under mechanical deformation, making them especially suitable for measuring subtle movements.

#### Crack engineering

Among the crack-based strain sensors developed, bio-inspired crack architectures deliver exceptional sensitivity (*GF* = 647) by exploiting controlled micro-fractures in conductive films^28^. However, the sensor exhibited a narrow operating strain range of up to 0.22%, limiting its suitability for large-deformation sensing. Therefore, although the sensitivity of the strain sensors based on the crack structure has been significantly improved, the maximum strain is limited. Zhou et al.^29^ and Wang et al.^30^ addressed this limitation. More specifically, Zhou et al.^29^ demonstrated that controlled fragmentation of single-walled carbon nanotube (SWCNT) papers embedded in PDMS enables ultrahigh gauge factors up to 10^7^ at 50% strain, establishing crack/fracture engineering as a key strategy for simultaneously achieving high sensitivity and high stretchability, whereas Wang et al.^30^ by tuning the carbon nanotubes (CNTs) film thickness, and afterwards the controlled network cracks forming “gaps, islands, and bridges” enabled high sensitivity with a maximum gauge factor of 87, a broad sensing range up to 100% strain, detection of strains as low as 0.007%, stable performance over 1500 cycles, and applications including pulse monitoring, speech and throat movement detection, hair touch sensing, music vibration detection, and tracking of wrist, finger, elbow, and knee movements. However, it is still mainly a lab demonstration. Issues such as long-term skin wear, sweat resistance, washability, manufacturing consistency, and real-world calibration would require more testing before practical wearable use. Wang et al. achieved a gauge factor of 35.75 at strains up to 45% using CNT-network crack propagation as the dominant sensing mechanism^31^, whereas Jin et al.^25^ focused on suppressing crack growth (by wrinkle structures) to improve durability, maintaining stable performance after 5000 stretching cycles at 50% strain with stretchability up to 800%. Together, these studies demonstrate that two contrasting design strategies have emerged: crack engineering, which enhances strain sensitivity through controlled crack propagation, and wrinkled structures, which improve mechanical robustness and durability by suppressing crack growth. Building on the concept of crack creation, Sun et al.^32^ used CNT and silver nanowires (AgNWs), creating a strong bond with the TPU substrate, and by pre-stretching it, they constructed a microcrack structure capable of combining characteristics of a wide working range (up to 171%) and ultrahigh sensitivity, up to *GF* 1.1 × 10^5^. Prestretching increased the number of microcracks, formed conductive pathways, and generated an ‘island–bridge’ structure. Liu et al.^33^ fabricated thickness-gradient conductive films using a self-pinning coffee-ring effect, enabling the formation of gradient wrinkle/crack structures with enhanced stretchability and strain sensitivity. However, the fabrication process relied on carefully controlled evaporation-induced gradient formation, which may limit reproducibility and large-scale manufacturing.

Subsequently, Park et al.^34^ demonstrated that controlling the depth of the crack in sputtered Platinum (Pt) films can significantly enhance the sensitivity of crack-based strain sensors. One of the main strengths of this work is the systematic investigation of the depth-dependent crack sensing behavior, providing important information about the relationship between crack morphology and electrical response. The sensor exhibited high sensitivity due to controlled crack propagation (*GF* = 16,000 at 2% strain), while the fabrication process, based on Pt sputtering and treatment with tensile strain, remained relatively simple and reproducible. However, the study also presents several limitations. The sensing range remained relatively limited compared to the sensors built in cracks or gradient structures mentioned above because an excessive crack opening can rapidly disconnect the conductive pathways. In addition, the use of sputtered Pt films may increase fabrication cost and reduce scalability for large-area wearable applications. Similarly, Wang et al.^31^ employed printable CNT/PDMS conductive networks to achieve highly stretchable sensors (up to 45%) with tunable sensitivity by controlling the number of CNT printing cycles (*GF* up to 35.75), while the fabrication process remains relatively simple and reproducible.

A simple, low-cost, and scalable manufacturing method based on the laser-induced graphene (LIG) fabrication process was introduced^35^. It also introduces self-healing crack behavior in the conductive graphite network, where cracks reopen and reconnect during deformation and recovery. However, the sensor is less sensitive than the crack-based systems mentioned above, with a maximum *GF* of 362 and some hysteresis, as well as reduced high-frequency response. In a subsequent study, ultrahigh sensitivity was achieved, with *GF* = 10,486, by creating an Ag nanowire (AgNW)-acrylate compound patterned using a novel ultrasonication-based patterning technique^36^. More specifically, they combine a CNT conductive crack layer on top of an electrospun TPU fibrous mat with pre-stretched crack engineering, achieving huge stretchability up to 300%, a very high gauge factor (*GF* > 83,982.8), and excellent durability (> 10,000 cycles) for wearable motion sensing solving the crack-sensor trade-off of high sensitivity and high stretchability. The key mechanism relied on the fibrils of CNT bridging adjacent crack islands during deformation, thereby preserving partial conductive pathways and enabling progressive amplification of resistance under large strains. Exceptional *GF* and stretchability are achieved, but the pre-fragmentation process may limit reproducibility and calibration stability. In a separate study, the authors described a strategy for forming parallel crack sensors that avoids the Poisson effect by preventing lateral crack reconnection^37^. More specifically, Liu et al. developed strain sensors based on ultrasonically patterned AgNW–acrylate composites, in which narrow conductive lines promote the formation of parallel cracks and suppress lateral crack reconnection. By adjusting the pattern dimensions, the sensors achieved a maximum gauge factor (GF) of 3.2 × 10⁷ at 1.5% strain and a GF of 10,486 at 8% strain in different sensor configurations. Controllable crack density was also achieved in another study by varying the nanocrystal loading in the conductive layer, resulting in high sensitivity (*GF* = 3405), the ability to detect high strains (up to 83%), and tunable multimodal sensing using a simple fabric-based process^38^. It also provides a practical wearable platform with breathable fabric, tunable strain and pressure sensing, and a simple dip-coating fabrication process. However, MXene oxidation/environmental stability may be a concern.

A unique microcrack fabrication method was introduced, based on a 3D-printing strategy for crack-based strain gauges, in which the sensing performance is controlled by the printing resolution and the groove structures formed during fused deposition modeling (FDM)^39^. Therefore, this work uses a 3D-printed TPU substrate coated with Pt, enabling self-aligned crack arrays to form along the printed groove valleys during prestretching. Another distinctive aspect is the focus on additive manufacturing scalability, reducing the need for complex micromachining and facilitating direct integration into wearable structures for healthcare monitoring applications such as pulse, voice, and motion sensing, but mainly optimized for small-strain healthcare monitoring rather than large-strain stretchability. In parallel, bioinspired dual-gradient and three-dimensional crack architectures demonstrated that engineered crack geometries can effectively balance sensitivity, stretchability, and durability for wearable sensing applications^40,41^. So, although *GF* is lower than some of the record-breaking crack sensors^29,36,37^, Liu et al. showed one of the best-balanced designs because it combines high sensitivity, large strain range, low hysteresis, and underwater functionality in a simple fabrication process^41^. A record-high study was inspired by the biological slit sensilla, the dual-gradient crack (DGC) sensor employed hierarchical thickness gradients to regulate progressive crack opening, achieving an ultrahigh gauge factor of 9 × 10^6^, an 80% sensing range, a low detection limit of 0.02% strain, and excellent durability over 22,000 cycles^40^.

Buckling engineering offers an alternative structural strategy, in which a stiff conductive film supported on a pre-stretched soft substrate forms a wrinkled or wavy structure upon strain release, as schematically illustrated in Fig. 3b. For example, anisotropic hierarchical V-groove/wrinkle structures enabled multidirectional strain detection with exceptional selectivity and tunable piezoresistive behavior^42^, while shape-reconfigurable crack systems introduced programmable and tunable sensitivity through controlled nanocrack formation on shape-memory substrates^42^. Lastly, shape-memory adaptive crack sensor (SMARTS) employed line-pattern-guided parallel nanocracks and thermally reconfigurable crack geometries to achieve an ultrahigh gauge factor of 2.7 × 10^9^ at 2% strain, an ultralow detection limit of 0.025% strain, a rapid response time of 0.025 ms, and stable operation over 20,000 cycles^43^. In addition to prestretching and controlled cracking, some studies combine prestretching with the large Poisson effect of conductive networks to further enhance stretchability and suppress wrinkles^44^. In this approach, the conductive network tends to undergo greater lateral contraction than the underlying substrate. Because this contraction is constrained by the substrate, transverse tensile stress develops in the network, resulting in a biaxial tensile state that suppresses wrinkling.

In general, crack engineering has become an effective approach for developing highly sensitive and durable strain sensors by using controlled crack propagation to improve the electrical response to mechanical deformation. However, excessive crack formation reduces stretchability, and sensor performance depends on parameters such as crack density, depth, morphology, and distribution. This dependence increases fabrication complexity and limits process reproducibility.

#### Auxetic architectures

Another aspect that researchers should consider for thin-film strain sensors is the intrinsic mechanical behavior of conventional materials, which contract laterally when subjected to tensile strain due to the Poisson effect (Fig. 3c). This phenomenon can compromise sensing performance and mechanical durability under large deformations. Auxetic materials (metamaterials) exhibit a negative Poisson ratio^45,46^, and have begun to regain attention in wearable and flexible strain-sensing applications. Unlike conventional materials, auxetic materials expand laterally when stretched, allowing a better mechanical response and a more uniform strain distribution^47^. For instance, “Kirigami patterns” allow out-of-plane deformation, increasing the sensor width, whereas in the normal configuration the width decreases^48^, resulting in improved sensitivity/stretchability^49,50^. Auxetic architectures based on repeated units (such as rectangular or re-entrant honeycombs, among others) can be engineered to increase sensitivity (Fig. 3d). For example, sensor performance was modulated to achieve an increase in sensitivity of up to 2^47^ or even 8^51^. This study^51^ further strengthens its real-world textile integration by maintaining performance after 10 washing cycles, highlighting the potential of textile-structural engineering for scalable wearable sensing. The various reported strain sensors, material systems, fabrication methods, and performance characteristics, including strain range, sensitivity, and targeted applications, are summarized in Table 1.

**Table 1:** Comparison of thin-film-based strain sensors for wearable applications

Ref.	Material system	Fabrication method	Maximum strain (%)	Maximum sensitivity (*GF*)	Demonstrated application
^19^	Crumpled graphene/ nanocellulose-PDMS composite	Vacuum-filtered nanopaper in PDMS	100	7.1	Finger-motion glove
^20^	Super-aligned CNT film/PDMS	Direct coating on PDMS	400	0.2	Motion glove
^25^	Silver flakes/PVDF	Electrospinning, stencil printing	800	1.52	E-textile electrodes
^28^	Carbon black (CB) ink/ glossy photo paper	Screen-printing followed by bending induced crack formation	0.22	647	Motion sensing, vibration detection, rotary encoders
^29^	Fragmented SWCNT paper/PDMS	Prefragmented SWCNT embedding	50	10^7^	Muscle sensing
^30^	Multilayer CNT films/PDMS	Prestretched CNT crack network, PDMS infiltration & prestretch	100	87	Pulse, throat movement, music vibration, finger/wrist/elbow/knee bending
^31^	Carbon nanotubes (CNTs)/ PDMS	Spray Deposition Modeling (SDM) printing of CNT ink on PDMS	45	35.75	Wearable human-motion monitoring, multidirectional strain sensing
^32^	CNTs & AgNWs/ Electrospun TPU-fibers mat	Electrospinning, vacuum-assisted filtration	171	1.1 × 10^5^	Sound/vibration detection, airflow, swallowing, finger/wrist bending, fist motion
^33^	SWCNT film/PDMS	Self-pinning coffee-ring effect (Gradient crack & wrinkle engineering)	150	161	Wearable vibration sensing
^34^	Pt thin film, PUA/PET	Pt thin-film sputtering, thin-film cracking and crack-depth control	2	16,000	Voice and force sensing
^35^	Graphite/PDMS or Ecoflex	Laser-induced graphite (LIG)	70	362	Pulse, finger bending, pressure sensing, robotic gripper feedback
^36^	CNT ink/TPU mat	Spray coating & prestretching	300	83,982.8	Large and subtle human-motion monitoring, pulse, phonation, joint bending, vibration detection
^37^	AgNW/acrylate/PDMS	Ultrasonicated AgNW lines	20	10,486	Pulse and throat-movement monitoring
^38^	MXene/CNC coating/ TPU nonwoven fabric	Dip coating	88	12,693	Full-range human motion, pulse, swallowing, respiration, walking, touch, E-skin pressure mapping
^39^	Pt thin film/TPU	3D-printed TPU, FDM & sputtering	1.67	184.88	Pulse, voice, hand motion, and customized finger-glove monitoring
^41^	Graphite/Ecoflex layer (Pre-cracked PDMS)	UVO-induced pre-cracking, 3D crack architecture & graphite/Ecoflex coating	100	1084.16	Human motion, pulse, speech/throat vibration, underwater sensing, wearable sensing in sweaty environments
^40^	Cr/Ag/Cr trilayer/ PDMS	Dual-gradient hierarchical cracks, thickness-modulated sputtering	80	9.2 × 10^6^	Facial expression sensing, finger motion, tremor detection, joint monitoring, wearable electronics
^43^	Sputtered Pt film/ PU/PLA SMP	Shape-memory crack tuning, Prestretch crack generation	N/A^a^	2.7 × 10^9^	Pulse monitoring, carotid/radial artery sensing, acoustic sensing, voice recognition, wearable electronics
^42^	Au thin film/ PDMS with V-groove microstructures	Template-transfer, biaxial prestretching	51	20,727.46	Multidirectional motion detection and directional strain sensing
^44^	Gold nanomesh (AuNM) transparent electrode/ PDMS	Grain-boundary lithography for Au nanomesh fabrication & transfer onto prestretched PDMS	N/A^a^	N/A^a^	Stretchable transparent electrodes, tactile sensors, flexible electronics
^46^	Graphene (CWPU/Graphene coating)/ 3D-printed TPU auxetic lattice	FDM 3D printing & dip coating	30	8.83	Wearable strain sensing, soft robotics
^47^	Eutectic gallium-indium (EGaIn) liquid metal electrodes/ Acrylic elastomer & silicone elastomer	Layer-by-layer fabrication, manual EGaIn filling, laminated assembly	50	1.61	Soft robotics, wearable deformation sensing, bending detection
^49^	Copper interconnects/ Polyimide	Laser-patterned polyimide	N/A^a^	N/A^a^	Stretchable electronics LED matrix display
^51^	rGO-coated warp-knitted auxetic fabric	Warp knitting & repeated rGO dip coating	40	3.4	Wearable motion monitoring, physiological sensing, smart textiles
^52^	PPy-coated elastic fiber with helical Cu wire	Vapor-phase polymerization of polypyrrole and manual helical winding	100	4	Textile-integrated strain sensing as shown in this study^322^

*Ref. [38]: Different CNC loadings resulted in different sensing performances. The table reports the maximum value for each metric: *GF* = 12,693 (MC0@p-NWF) and strain-sensing range = 88% (MC1.0@p-NWF). These maximum values correspond to different sensor configurations.

*Ref. [46]: Multiple lattice geometries were evaluated in the original study. Only the maximum reported value for each metric is presented in the table: *GF* = 8.83 (TR) and strain-sensing range = 30% (HN). These maximum values correspond to different sensor configurations.

N/A^a^: Not reported in the original study.

*GF* gauge factor, *PDMS* polydimethylsiloxane, *CNT* carbon nanotubes, *PVDF* poly(vinylidene fluoride), *SWCNT* single-walled carbon nanotube, *AgNW* silver nanowire, *TPU* thermoplastic polyurethane, *Pt* platinum, *PUA* polyurethane acrylate, *PET* polyethylene terephthalate, *Gr* graphene, *MXene* two-dimensional transition metal carbide/nitride, *CNC* cellulose nanocrystal, *Cr* chromium, *Ag* silver, *Au* gold, *PU* polyurethane, *PLA* polylactic acid, *SMP* shape memory polymer, *AuNM* gold nanomesh, *CWPU* conductive waterborne polyurethane, *FDM* fused deposition modeling, *EGaIn* eutectic gallium-indium, *Cu* copper, *PI* polyimide, *PPy* polypyrrole.

Despite advancements in sensitivity/stretchability, film-based strain sensors face important limitations with respect to textile integration. Thin-film sensors work well when sensing is needed at a specific, localized body site (e.g., a finger joint, wrist, elbow, or knee), but they become less suitable when the goal is to create large-area or whole-garment sensing systems because the continuous film can compromise the textile properties of the garment. In addition, most thin-film devices are fabricated on continuous polymeric substrates that can be attached or laminated to garments; however, this approach may reduce key textile characteristics such as air permeability, moisture transport, softness, and wearer comfort. Therefore, achieving seamless integration while preserving the inherent flexibility, breathability, and comfort of textiles remains a critical challenge for the widespread adoption of thin-film-based wearable sensors.

While the previous section focused on structural design strategies for thin-film strain sensors, it is also important to study how these behaviors translate to the yarn level. An important study by Cuthbert et al.^52^ investigated both auxetic structural design and yarn characteristics, providing a useful link between macro-structural auxetic behavior and yarn-based mechanical response. A schematic illustration of an auxetic yarn in the relaxed and stretched states is provided in Fig. 3e. In that study, they demonstrated that helical auxetic yarn architectures can overcome the conventional sensitivity limit of capacitive strain sensors, achieving gauge factors up to 4 through geometry-driven electrode interaction.

## Fiber- and yarn-based strain sensors

Fiber-based electronic devices (FEDs) have emerged as promising platforms for wearable systems because they are lightweight and have seamless textile integration capabilities. To achieve mechanical compliance and comfort in real garments, efforts have focused on conductive fiber and yarn-based systems that offer mechanical flexibility and easy integration into wearable garments^53–56^. Recent reviews have identified FEDs as a promising platform for wearable systems due to their flexibility, lightweight nature, and seamless integration into textiles^57^.

Fiber- and yarn-based strain sensors represent probably one of the important classes of wearable devices^58–60^ with a wide range of materials and fabrication methods that have been explored. In fiber-based electronics, the core material must have intrinsic flexibility and stretchability to accommodate repeated mechanical deformation and be compatible with the conductive layer^57,61–63^. The mechanical mismatch between the substrate and the conductive layer can induce interfacial instability and degrade electrical performance under stress^64^. For example, TPU is often used for stretchability exceeding 300%^65–67^. Other commonly used stretchable materials include spandex (especially suitable for textile applications)^68,69^, polyurethane (PU) fibers^66,70^, PDMS^71^, and thermoplastic elastomers (TPEs)^72,73^. Various strategies have been developed to enhance the performance of fiber-based strain sensors, including surface coating techniques, structural engineering, and the design of novel fiber architectures.

### Coated conductive fibers

**Fig. 4:**
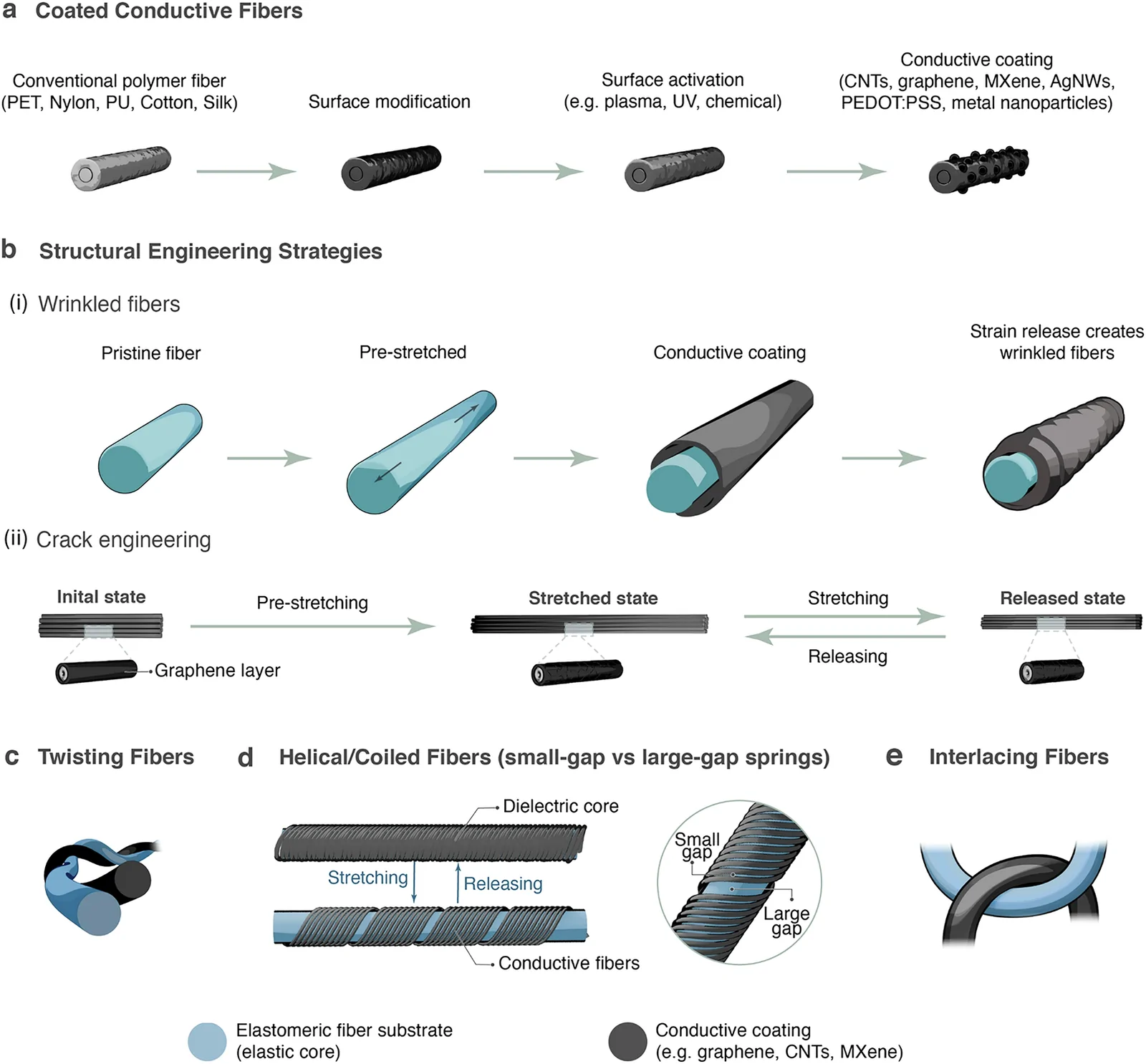
Schematic representation of Fiber- and Yarn-Based Strain Sensors. **a** Coated conductive fibers: Conductive coatings deposited on fiber substrates impart electrical functionality while preserving flexibility and stretchability. Compiled from refs. ^77,78,81^. **b**(i) Structural and crack engineering: Wrinkled or wavy conductive structures are formed by depositing conductive materials onto prestretched dielectric fibers, where release of the prestrain generates surface wrinkles. Adapted from ref. ^70^, with permission from John Wiley & Sons. **b**(ii) In contrast, prestretching of coated fibers induces controlled microcracks in the conductive layer, enhancing strain sensitivity and leading to high gauge factors. Adapted from ref. ^79^ Copyright 2023, with permission from Elsevier. **c**–**e** Fiber assembly engineering: Structural strategies such as twisting (adapted from ref. ^78^ with permission from the American Chemical Society), helical architectures (adapted from ref. ^82^; Copyright © 2019 American Chemical Society), and interlacing (adapted from ref. ^83^ under the terms of the CC BY-NC 4.0 license; © 2021 The Author(s), published by the Royal Society of Chemistry) enable enhanced stretchability and tunable electromechanical performance in fiber-based sensors.

Conductive-coated fibers constitute a simple and effective approach for fabricating fiber-based strain sensors for wearable applications. By depositing conductive materials onto elastic fiber substrates, improved flexibility and sensing performance can be achieved. For example, highly elastic PU can be used as a core fiber, and additional polyester can be used as an insulating polymer that offers mechanical support^74^. Then, conductive yarns are manufactured through several methods, including metal-infused polymers or the addition of conductive coatings to conventional yarns. As schematically illustrated in Fig. 4a, a conventional polymeric fiber is transformed into a conductive fiber through successive coating and functionalization steps. Through surface activation^75^ and subsequent deposition of a conductive coating^76^, non-conductive fibers can achieve improved interfacial adhesion and uniform conductivity under large strains^77^. However, fabrication processes often involve multiple coating, wrapping, or polymerization steps, which may increase manufacturing complexity and limit large-scale production. In addition, maintaining long-term interfacial adhesion between the conductive sheath and the elastic core under repeated deformation remains a significant challenge. A simple, scalable approach involves fabricating a sheath-core yarn with an elastic rubber or spandex core, which is wrapped with cotton or CNT yarn. The yarn is then coated with CNTs and subjected to in situ polymerization of polypyrrole (PPy) on its surface^78^. The resulting sheath-core yarn functions as a portable strain sensor and demonstrates an ultrahigh strain sensing range of up to 350%.

### Structural engineering strategies (e.g., pre-stretching, crack engineering, wrinkle engineering)

Beyond coating, metal-nanowire composites can be used as scaffolds on stretchable fiber for high-performance mechanical sensing. As shown in Fig. 4b(i), pre-strained fibers can develop wrinkled multiscale microstructures upon release, enabling high stretchability up to 400%^70^. For fiber sensors that exploit surface microstructuring, prestretch-programmed microcracks yield ultra-high sensitivity and are ready for use in textiles after repeated dip coating^79^. Extending these prestretching-based design principles to conductive-coated fibers, researchers have also stretched conductive-coated fibers after coating deposition, as shown in Fig. 4b(ii). Cracks can provide high sensitivity by disrupting conductive pathways, while wrinkles enhance stretchability by accommodating deformation. This synergistic structure enabled a wide strain range of 0–200%, a high *GF* of 1344.1, and durability over 10,000 cycles, making it suitable for textile-based human motion monitoring^80^.

### Structural fiber architectures (helical/coiled, twisted, braided, interlaced)

In addition to surface engineering methods such as prestretching and crack or wrinkle formation, the overall fiber architecture is critical to the mechanical compliance and sensing performance of fiber-based strain sensors. Helical, coiled, twisted, and interlaced fiber structures can accommodate large deformations through geometric reconfiguration, thereby improving stretchability while preserving the integrity of the conductive network. Structural engineering strategies have been widely employed to tailor the electromechanical response of fiber-based strain sensors. Pan et al.^81^ embedded a textile-first design, polydopamine (PDA)-primed braided PET on an elastic core with in-situ PPy that chemically secures the conductor and uses braid-angle reconfiguration to decouple sensitivity from stretchability, delivering a *GF* of 51.2 over a strain range of 0–40% and a *GF* of 28 over a strain range of 40–105%, with abrasion-tolerant adhesion and direct weavability for practical wearable integration, offering a proposal to create a special textile structure, simultaneously with a bonding mechanism between fibers and conductive compounds. Lu et al. leverage the intrinsic helical microgeometry of a rubber-core/PP-spring yarn with a CNT sheath to create hierarchical (double-level) gaps, as shown in Fig. 4c^82^. This design exhibits a unique structure that can amplify minute strains while avoiding crack failure, yielding ultralow detection limits due to the design of “double-leveled helical gaps”. This novel microstructure was achieved using a highly elastic rubber fiber core tightly wound with a continuous spring-like polypropylene fiber shell. In contrast to previous textile sensors that depend on geometric deformation or fragmentation of the conductive network, Lee et al. took a different approach^83^ by using two different yarns, selecting materials with resistance-change properties: aluminum-coated and carbon fiber. Their method depends on changes in the contact area between the two materials, as schematically illustrated in Fig. 4d. They treated the textile knit as an array of resistive random-access memory (RRAM) cells. When mechanical deformation is applied, the fabric acts as a distributed, memory-like electronic network, with each contact point functioning as a tiny metal-insulator-metal (MIM) junction, rather than as a simple resistive thread. Although these sensing architectures improve device performance, practical implementation also requires effective integration into textile and garment platforms. The key characteristics of fiber- and yarn-based strain sensors are summarized in Table 2.

**Table 2:** Comparison of yarn/fiber-based strain sensors for wearable applications

Ref.	Material system	Fabrication method	Maximum strain (%)	Maximum sensitivity (*GF*)	Demonstrated application
^74^	Graphene (RGO-coated fiber), Polyurethane (PU) core with polyester (PE) helical yarn	Dip-coating of GO on plasma-treated elastic yarn followed by HI reduction	100	10	Human motion monitoring, speech recognition, pulse/breath monitoring, robotic motion sensing
^76^	Silver-coated polyester fiber, polyester fiber	Electrical conduction through metallic Ag coating, Continuous electrodes plating & electroplating	N/A^a^	N/A^a^	Conductive textiles/electronic fibers
^78^	CNTs & PPy, Rubber thread core & cotton sheath	Helical deformation, CNT-coated cotton yarn wrapped around rubber thread, CNT dip coating, PPy polymerization	350	5.11	Human motion, writing, drinking/swallowing
^70^	AgNWs & WPU binder, Commercial PU fiber	AgNW/WPU ink coated on 200% prestrained PU fibers, release forms multiscale wrinkles	400	N/A^b^	Pressure/bending sensing, finger-motion detection, pressure mapping
^79^	rGO nanosheets with PDA/PVA layers, PU yarn	Crack open–close piezoresistive response in rGO sheath, PVA coating, rGO dip coating, PDA self-polymerization	90	131.8	Rehabilitation monitoring, gait detection, chewing, palm/elbow/knee sensing
^80^	MWCNT ink, PU yarn	Wrinkle-assisted crack opening and tunneling resistance increase, CNT coating followed by prestretch/release treatment	200	1344.1	Finger, wrist, knee, pulse, respiration, blinking, speech monitoring
^81^	PPy, Braided PET fibers around elastic polymer core	PDA-coated PET fibers braided around elastic core, PPy in situ polymerization	105	51.2	Motion monitoring, facial expression, breathing, speech detection, wearable heating
^82^	CNT-coated helical fiber, elastic rubber core with PP helical shell	Dip-coating/ultrasonic absorption of CNT ink onto spring-like fiber	200	6.14	Wearable motion and physiological sensing
^83^	Carbon fiber and Al-coated yarn, Knitted textile yarn with cotton support	Knitting of Al-coated and carbon fiber yarns	43	0.9	Wearable motion and joint sensing
^65^	CNT & AgNW hybrid network, porous EGaIn/TPU fiber core	Wet-spinning of EGaIn/TPU fiber, CNT and AgNW dip coating	500	7336	Gesture recognition, Morse-code communication, motion monitoring
^95^	Ti_3_C_2_T_*x*_ MXene, cotton sheath & spandex core	Contact-resistance change under compression through MXene dip coating of cotton roving followed by friction spinning	100	N/A^b^	Motion, touch, breath monitoring, Morse-code communication
^66^	AgNWs & CNT conductive core, PU nanofiber scaffold & elastic TPU core	One-step conjugated electrospinning, electrospraying, and twisting onto elastic core	160	1010	Strain, pressure, bending, speech, swallowing, facial motion, Morse code, pressure mapping
^323^	EGaIn liquid metal particles, TPU	Heterostructure printing, twisting, sintering	200	N/A^a^	Digital apparel, healthcare monitoring
^100^	PVDF or PCL porous domains with COC cladding	Preform-to-fiber thermal drawing	N/A^a^	N/A^a^	Tunable porous morphology (not stretchability-focused), Multifunctional porous fibers, ionic transport, smart textiles
^101^	Gallium / Galinstan liquid metal, SEBS thermoplastic elastomer	Multimaterial thermal drawing with liquid metal-filled channels	500	1.8	Stretchable interconnects, strain/pressure sensors, smart textiles, soft robotics
^102^	Liquid metal (Galinstan) dual helical microchannels, SEBS elastomer	One-step spiral thermal drawing followed by LM injection	1000	1	Wearable strain/pressure sensing, smart textiles, motion monitoring and Morse-code communication
^103^	Carbon-black-loaded polyethylene (cPE), SEBS thermoplastic elastomer	Multimaterial thermal drawing, fiber attached/integrated onto large textile mat	4	N/A^b^	Large-area pressure mapping, posture/motion monitoring, gymnastic mat, health monitoring
^104^	Liquid metal microchannel transmission lines, SEBS thermoplastic elastomer	Thermal drawing of elastomeric fibers with embedded liquid metal conductors	100	N/A^a^	Electronic textiles, soft robotics and wearable sensing
^105^	CNT-based thermoplastic elastomer nanocomposite, SEBS thermoplastic elastomer	Thermal drawing of CNT-SEBS multimaterial nanocomposite fibers	195	94.2	Soft robotics, pressure sensing and wearable mechanical sensing
^106^	EGaIn liquid-metal embedded elastomer, elastomeric polymer (LMEE matrix)	Thermal drawing of liquid-metal-embedded elastomer preforms	925	0.96	Embedded liquid-metal conductive and dielectric domains enabled ultrastretchable and mechanically stable multifunctional fibers
^108^	Copper microwire electrode, SEBS or COCe elastomeric fiber	Thermal drawing followed by post-draw buckling or twisting/annealing	50	N/A^b^	Stretchable electronic textiles and optical communication fabrics
^107^	Copper microwires, semiconductor memory devices, digital temperature sensors	Thermally drawn polymer fiber architecture	N/A^a^	N/A^a^	Physiological monitoring and activity inference
^109^	Helically structured copper microwire electrodes, ECOC elastomeric fiber	Thermal drawing with foldable interposer integration	67.1	N/A^a^	Helical metallic interconnects enabled elastic thermally drawn fiber computers integrating sensing, processing and wireless communication

N/A^a^: Not reported in the original study, N/A^b^: The device was primarily designed for pressure sensing.

*RGO* reduced graphene oxide, *PU* Polyurethane, *PE* polyester, *CNT* carbon nanotube, *PPy* polypyrrole, *AgNW* silver nanowire, *WPU* waterborne polyurethane, *PDA* polydopamine, *PVA* poly(vinyl alcohol), *PI* polyimide, *PP* polypropylene, *MWCNT* multi-walled carbon nanotube, *PET* polyethylene terephthalate, *Al* aluminum, *EGaIn* eutectic gallium-indium, *Ti*_*3*_*C*_*2*_*T*_*x*_* MXene* titanium carbide MXene, *PVDF* poly(vinylidene fluoride), *FOC* fiber optic cable, *LM* liquid metal, *Galinstan* Gallium-indium-tin alloy, *SBS* styrene-butadiene-styrene, *HTLSP* high-temperature liquid silicone polymer, *ECOC* elastomer-coated optical core.

## Scalable manufacturing and fabrication techniques

Despite the remarkable sensing performance reported for many laboratory-scale strain sensors, the lack of scalable fabrication methods remains a major barrier to their large-scale deployment. Therefore, greater attention should be given to scalable fabrication approaches that facilitate the practical implementation of fiber-based strain sensors and their translation to industrial production.

**Fig. 5:**
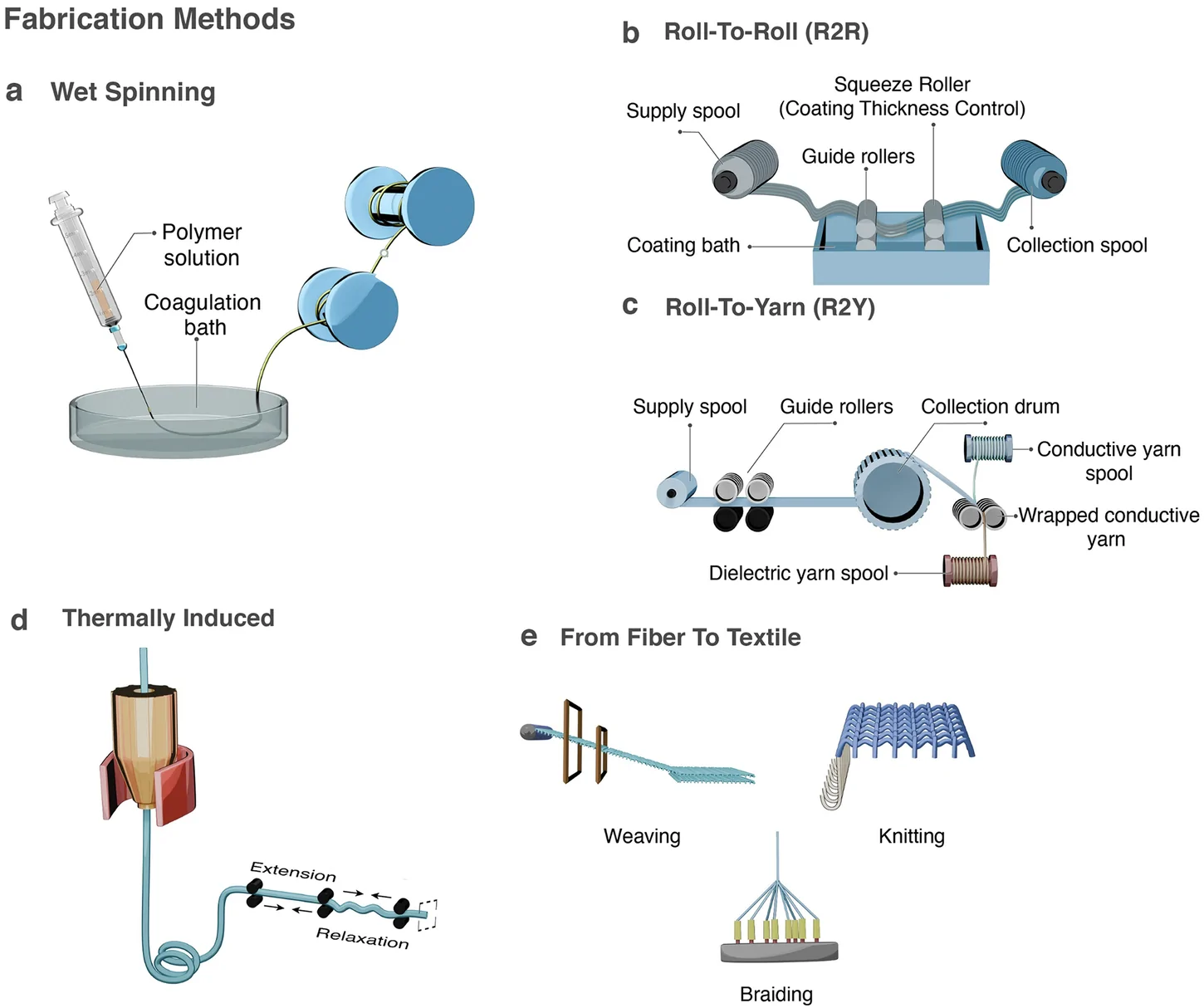
Schematic representation of scalable manufacturing approaches for fiber-based sensors. **a** Wet spinning: Fabrication of conductive fibers through solution-based spinning processes. Adapted from ref. ^65^. **b**, **c** Roll-to-roll/ Roll-to-yarn processing: Continuous large-scale fabrication of conductive fibers and flexible sensors. Adapted from ref. ^94^ ACS Applied Materials & Interfaces 15, 10994–11003 (2023). Copyright 2023 American Chemical Society. **d** Thermally induced fabrication: Formation of conductive or porous structures via thermal treatment and temperature-driven processes. Adapted from refs. ^107^, © 2022 The Authors. Advanced Materials published by Wiley-VCH GmbH. **e** Textile integration: Incorporation of functional yarns into wearable systems through weaving, knitting, and braiding techniques. Adapted from refs. ^335^, with permission from Elsevier.

Among the various scalable fabrication approaches developed for fiber-based strain sensors, wet-spinning is one of the most versatile and widely adopted techniques, with various designs and properties, including, among others, core-shell fibers, hierarchical structures, and hollow fibers^84^. A simplified schematic is illustrated in Fig. 5a, in which a polymer solution is extruded into a coagulation bath to form continuous fibers. Due to the relatively simple process, high potential for scalability, and compatibility with various conductive nanomaterials and polymers^85–87^, wet spinning facilitates the continuous production of functional fibers with tunable mechanical and electrical properties^88^. Furthermore, the process facilitates the incorporation of conductive fillers, such as carbon nanotubes, graphene, MXenes, and metallic nanomaterials, within polymer matrices, which makes it particularly attractive for wearable sensing applications^89^. Several specialized reviews have summarized wet spinning principles, suitable materials, and inherent challenges, and have compared them with other spinning techniques used to develop flexible conductive fibers^89,90^. Depending on the composition of the spinning dope and coagulation conditions, the resulting fibers may exhibit a dense and homogeneous cross-section or a porous internal structure^91^. This morphological control plays a crucial role in determining the mechanical, electrical, and sensing properties of the final fiber-based device. For example, a highly stretchable fiber-shaped strain sensor with ultrahigh sensitivity was obtained by combining a hierarchically porous liquid metal-polymer core (EGaln/TPU) with a hybrid AgNW/CNT^65^. This dual-layer design synergistically combines stretchability, a unique feature of the porous core structure, with sheath conductivity/sensitivity, maintaining high elasticity up to 500%. The porous liquid-metal TPU core further enhanced mechanical deformability, while the fiber geometry enabled textile integration for wearable motion sensing applications.

Beyond solution-based spinning techniques, continuous manufacturing platforms have been developed to improve production throughput and facilitate industrial-scale fabrication. Among these, roll-to-roll and roll-to-yarn processes (Fig. 5b, c) have emerged as promising strategies for large-scale fabrication of flexible conductive fibers^92,93^. For example, Zou et al.^94^ fabricated MXene/cotton/spandex core yarns through industrial friction spinning, demonstrating a continuous and scalable route to MXene-based wearable electronics. Unlike wet-spun conductive fibers, the conductive component was introduced through surface coating prior to yarn formation, enabling textile-compatible manufacturing and facile integration into a wearable sensing platform. Electrospinning has also been used for the fabrication of conductive yarns^95,96^. For example, Dai et al. used a one-step conjugated electrospinning and electrospraying process to produce AgNW-embedded coaxial nanofiber-coated yarns with a hierarchical porous conductive sheath surrounding an elastic core fiber^66^. In that study, lightweight coaxial nanofiber-coated yarns were constructed that could be attached directly and conformally to curved human skin and integrated into textile sensing arrays using a continuous one-step process. More recently, Lee et al. demonstrated a scalable heterostructure-printing approach for fabricating meter-scale conductive fibers composed of liquid-metal particles embedded in a TPU matrix. Subsequent twisting and sintering improved the mechanical robustness of the fibers and enabled their integration into intelligent digital apparel^97^.

Thermally induced phase separation (TIPS) is among the most widely investigated approaches in this field, relying on temperature-driven soldification process, enabling the formation of fibrous structures with tunable morphology and porosity^98,99^. Figure 5d shows a macroscopic preform containing the desired material, heated above its softening temperature, and continuously drawn into a fiber through a series of extension and relaxation stages. A key advantage of thermal drawing lies in its ability to produce complex internal architectures and allows the process to be adapted to generate porous structures. For example, Grena et al. demonstrated that thermal drawing can be combined with phase separation to generate spatially selective porous domains within multimaterial fibers^100^. Although stretchability may be limited, depending on the underlying electronics, thermally induced fabrication offers attractive scalability for kilometer-long production in wearable systems. High stretchability can also be achieved by directly encapsulating materials such as liquid metals within the fiber. Building on these efforts, they demonstrated a superelastic multimaterial that allowing for strain up to 500% and pressure detection, while differentiating between them (pressure and strain) and compensating for thermal or other drifts^101^. Zhang et al.^102^ shifted the focus to structural optimization of liquid-metal fiber architectures. By introducing helical conductive channels within thermally drawn elastomer fibers, they improved mechanical compliance, sensing stability, and ultrahigh stretchability while reducing directional sensing errors commonly observed in straight-channel liquid-metal fibers. Leber et al.^103^ advanced thermally drawn fiber sensors from simple stretchable conductors toward distributed large-area pressure sensing. Their fiber design used carbon-black loaded polyethylene electrodes embedded in a compressible poly[styrene-*b*-(ethylene-co-butylene)-*b*-styrene] (SEBS) matrix, where applied pressure collapsed the hollow structure and selectively brought the electrodes into contact. This enabled both pressure-level detection and localization along meters-long fibers, reducing the need for dense sensor arrays. The work is technically strong because the fiber geometry was supported by finite-element analysis and demonstrated on a large gymnastic mat for posture and motion monitoring. However, pressure-level detection relies on discrete electrode-contact events, which may limit the resolution of pressure quantification. In addition, resistance changes due to temperature, axial deformation, or complex bending require compensation and may reduce reliability in real-world textile use. Therefore, although the study provides a scalable and elegant route toward large-area pressure-sensing textiles, more work is needed to validate durability, washability, bending tolerance, and long-term stability under realistic wearable conditions. A follow-up study^104^ represents a major conceptual advancement because it transforms thermally drawn fibers from passive resistive sensors into actively interrogated distributed sensing systems. In particular, the use of liquid-metal conductors embedded within elastomeric dielectrics enabled the high deformability while maintaining transmission-line functionality, overcoming limitations of conventional rigid metallic reflectometry probes. Furthermore, integrating a single sensing line into large-area fabrics significantly reduced the number of electrical interconnects required compared to traditional sensor-array architectures. Building on their earlier work on thermally drawn superelastic multimaterial fibers, the same group progressively advanced the platform from stretchable fiber conductors to more application-specific mechanical sensors. More specifically, they further extended the platform by engineering CNT-based thermoplastic elastomer nanocomposites compatible with thermal drawing^105^. This allowed the electrical resistivity and piezoresistive response of the fibers to be tuned through processing temperature, enabling targeted fiber designs for bending, pressure, and stretch sensing. A major breakthrough stretchability was achieved up to 925%, completely transforming thermal drawing from a mostly rigid multifunctional fiber platform into an ultrastretchable electronic fiber platform using liquid-metal elastomer composites^106^.

In parallel, Marion et al. sought to solve a very specific problem in electronic textiles: how to make a single fiber that is highly conductive, such as a metal wire, and elastic, such as a textile yarn^107^. Therefore, they produced thermally drawn elastomer fibers with an embedded solid metal electrode, yielding stretchable, conductive fibers for electronic fabrics. In a second design, they made a fiber with an off-center metal wire and then twisted the fiber. After being twisted and annealed, the wire became a helix inside the elastomer. When stretched, the helix unwinds or changes pitch. The stability trade-off in thermally drawn fibers is achieved by embedding buckled or helical solid-metal electrodes within elastomeric claddings, enabling highly conductive and stretchable interconnects with minimal resistance variation. In contrast, strain-sensing fibers intentionally exploit resistance or capacitance changes under deformation to transduce mechanical strain. More recent studies demonstrate a transition from passive conductive textiles to computational textile systems capable of distributed sensing, memory storage, communication, and ML inference. In a breakthrough paper, instead of concentrating computation on a single chip, researchers distribute functionality along the fiber, reflecting broader trends in computing and distributed systems^108^. This work is particularly interesting, as biological systems are distributed, with human skin containing sensory receptors spread across the body rather than centralized in one location. By embedding electronics throughout the textile, the researchers move closer to biomimetic computing architectures, where sensing and processing are integrated into the material itself. This paper demonstrates a path to transformative wearable technology that can be mass-manufactured, remain flexible, and integrate with existing textiles. Conceptually, this transforms a fiber from a passive conductor to an intelligent communication “bus”. However, the sensing modality is primarily a temperature-based activity interface, and the demonstration remains a proof of concept rather than a fully general wearable artificial intelligence (AI) system. Some practical challenges, such as long-term washability, power-supply integration, thermal management, data security, and repairability, remain unresolved. This paper demonstrates feasibility, but there is no large-scale consumer readiness yet.

In a follow-up study, they developed a fully functional computer embedded within a flexible textile fiber, containing sensors, digital memory, microcontrollers, communication systems, and power capabilities^109^. Moreover, they connect the devices using helical copper microwires (spring-like), which can stretch elastically. They also show that the fiber can stretch over 60%, bend, and maintain its functionality after washing. This work represents an important step toward ambient and embodied computing, in which intelligence becomes integrated directly into everyday materials rather than existing as separate electronic devices. Beyond enabling multimodal sensing architectures, recent perspectives emphasize that progress in rheology, fluid dynamics, and materials compatibility is now driving the transition of thermally drawn fibers from laboratory demonstrations to industrially manufacturable smart systems^110^. Notably, alongside the technological advances demonstrated in this work, the integration of machine learning (ML) within wearable textiles introduces ethical considerations regarding privacy, surveillance, and data ownership that warrant further discussion. These studies laid the foundation for later developments in soft and stretchable electronic fibers, including distributed pressure sensors, liquid-metal transmission lines, and nanocomposite-based wearable sensing systems.

In conclusion, continued advances in multifunctional materials, fiber-based electronics, and textile manufacturing (e.g., weaving, knitting, and braiding) (Fig. 5e) are expected to accelerate the translation of intrinsically stretchable strain sensors from proof-of-concept demonstrations to next-generation smart textiles and personalized healthcare systems.

## Quantitative and qualitative assessment of reported wearable sensors

Quantitative and qualitative data were extracted directly from the text or tables from each study. Quantitative data include sensitivity, stretchability, and number of mechanical cycles, whereas qualitative data include textile compatibility and ease of integration.

To enable comparison across studies reporting different quantitative performance metrics, raw values were converted into standardized scores ranging from 1 (lowest performance) to 5 (highest performance). Scores were assigned based on predefined quantitative thresholds for each metric. The extracted performance metrics were subsequently assessed using a five-point ordinal scale: Low (1), Low to Moderate (2), Moderate (3), Moderate to High (4), and High (5). A detailed comparison of sensitivity, stretchability, durability, textile compatibility, and ease of fabrication across representative studies is provided in the Supplementary Information. The quantitative scoring thresholds and qualitative evaluation criteria used for the comparative assessment are also provided. Each study was evaluated independently. Average scores for each criterion were calculated using only studies with available and directly comparable numerical scores. Entries reported as ‘NR’ (not reported), ‘N/A’ (not applicable), or ‘Not directly comparable,’ as well as excluded studies, were omitted from the corresponding average calculation. Consequently, the number of studies included in the average may vary across criteria.

**Fig. 6:**
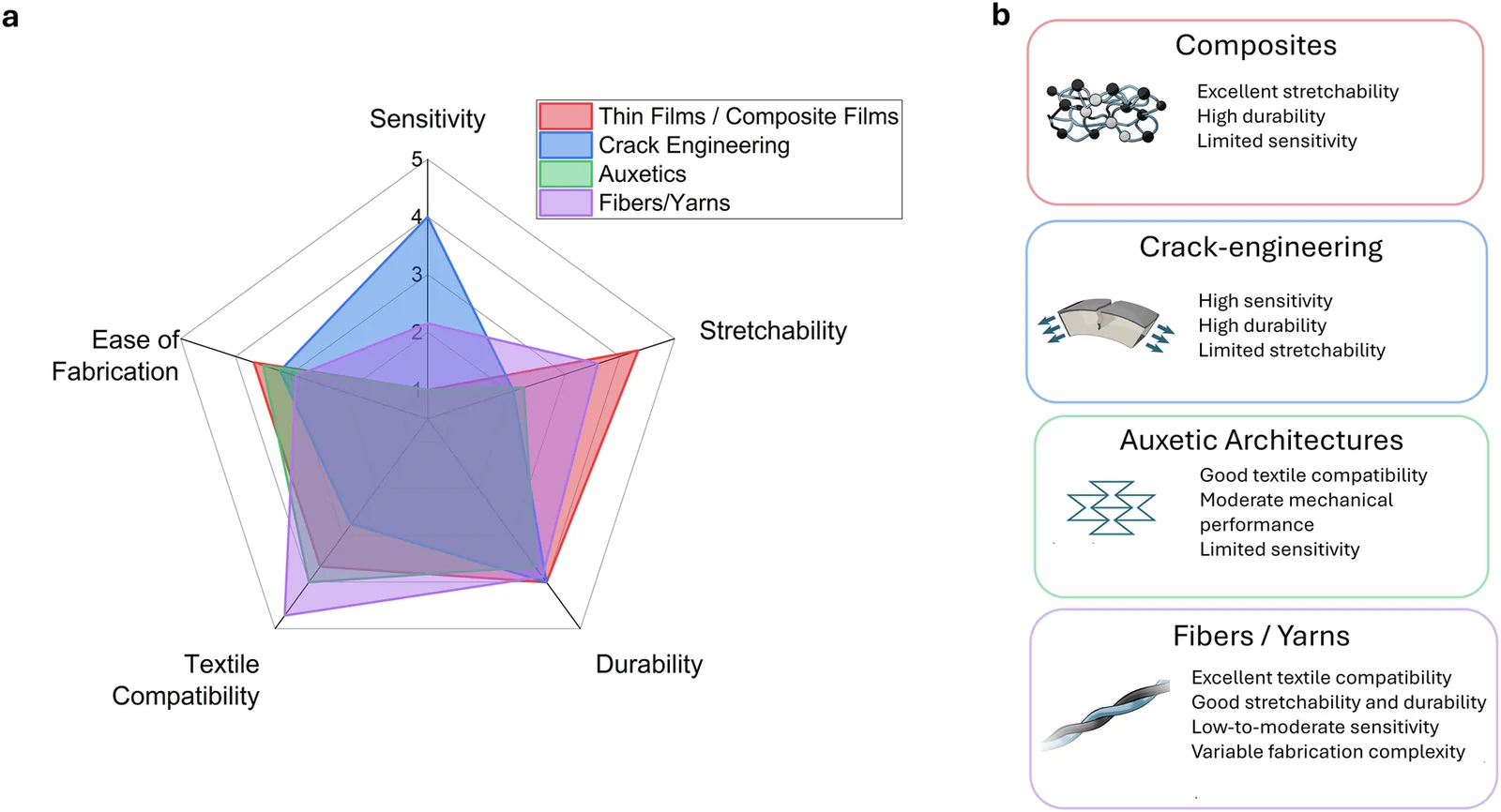
Comparative synthesis of reported wearable sensors. **a** Multicriteria performance comparison of representative design strategies based on sensitivity, stretchability, durability, ease of fabrication and textile compatibility. **b** Summary of the key advantages and limitations associated with major design strategies. Note: Average scores were calculated using predefined 1–5 scoring criteria for sensitivity, stretchability, durability, ease of fabrication, and textile compatibility. Only studies with available and directly comparable data for a given criterion were included in the corresponding average. Entries designated as N/R (not reported) or N/A (not applicable), as well as values that were not directly comparable, were excluded from the calculation. Consequently, the number of studies (n) contributing to each average differed among criteria; the corresponding n values are reported in the Supporting Dataset.

The results are visualized in Fig. 6a, highlighting trends, trade-offs, and design differences among the various sensor strategies. However, because the reviewed studies used different experimental conditions, materials, device architectures, and performance evaluation protocols, the analysis is intended as a descriptive comparison rather than a formal quantitative meta-analysis. Some key summaries are also provided in Fig. 6b. In general, the evolution of wearable stretchable sensors reflects a persistent trade-off between sensitivity, mechanical compliance, durability, and integration scalability. *Crack-engineered systems* often achieve high to ultrahigh gauge factors through localized strain amplification and generally demonstrate high durability, but their working strain ranges tend to be more limited. Conductive composite systems generally offer excellent stretchability and high durability, together with relatively accessible fabrication approaches, but typically exhibit low sensitivity. *Auxetic and structurally engineered architectures* represent promising intermediate strategies that exploit geometry-driven deformation while providing good mechanical adaptability and strong compatibility with textile integration, although their sensitivity is generally limited. *Fiber- and yarn-based sensors* demonstrate the highest textile compatibility and wearability while maintaining good stretchability and durability. Their intrinsic integration within fabrics makes them particularly attractive for smart textiles and long-term wearable monitoring, although sensitivity may vary depending on the sensing mechanism and fiber architecture.

In conclusion, the comparative analysis presented in this review highlights the trade-offs among current design strategies and provides a framework for selecting appropriate material and structural approaches based on targeted wearable applications. Optimal wearable sensor design requires balancing multiple factors. However, no single approach achieves ultrahigh sensitivity, a large strain range (stretchability), long-term durability, low fabrication complexity, and seamless textile integration simultaneously.

## Structural engineering strategies for stretchable circuits and interconnects

Although wearable sensors are central to wearable electronics, their practical implementation often requires integration into stretchable conductive circuits capable of signal transmission, sensing, and power delivery. Consequently, considerable effort has been devoted to the development of stretchable conductive circuits and flexible printed circuit boards (PCBs), which serve as a platform for signal processing, power management, and communication. Despite their close integration within wearable electronic systems, wearable sensors and stretchable circuits/interconnects have distinct functional roles and design objectives, as summarized in Table 3. Traditionally, electronic circuits have been implemented on PCBs^111^, which provide mechanical robustness and high electrical reliability through defined copper traces, but have limited ability to accommodate bending or deformation. In addition, PCBs are incompatible with the stretchable, nonuniform nature of textile substrates, making flexible interconnect strategies essential. To improve mechanical compliance, flexible printed circuit boards (FPCBs) were subsequently developed by fabricating metal conductors on thin polymer substrates, allowing bending and folding while maintaining conventional circuit performance^112^. However, because the metallic conductors remain intrinsically rigid, the FPCBs exhibit limited tolerance to tensile stress^113^. The use of flexible substrates, such as polyimide (PI), TPU, or silicone elastomers, has led to the development of alternative approaches for smart textile conductive circuits^114^, integration methods including the application of thin, flexible circuit films laminated to textiles in specific patterns^114–116^.

**Table 3:** Comparison of the functional roles and design objectives of wearable sensors and stretchable circuits/interconnects

Aspect	Wearable sensors	Stretchable circuits and interconnects
Primary function	Convert external mechanical, chemical, or physiological stimuli into measurable electrical signals	Enable power delivery, signal transmission, and system-level integration
Design objective	Maximize sensitivity, responsiveness, and sensing accuracy	Maintain stable electrical performance under mechanical deformation
Role of structural engineering	Enhance transduction mechanisms and strain-induced signal variations	Accommodate strain while preserving electrical continuity and signal integrity
Representative strategies	Crack engineering, wrinkle engineering, pre-strain engineering, auxetic structures	Crack engineering, wrinkle engineering, pre-strain engineering, serpentine interconnects
Key performance metrics	Sensitivity, gauge factor, stretchability	Conductivity retention, resistance stability, durability, stretchability

Subsequently, the growing demand for electronics that can conform to soft, deformable, and dynamic surfaces has driven the development of stretchable electronics, which are designed to maintain a high tensile deformation through geometric design or intrinsically stretchable materials^117,118^. That was driven by the need for systems to have skin or tissue-like mechanical properties, including low stiffness modulus and high stretchability, and to be used to create intelligent technologies for several applications in health monitoring and human–machine interaction (HMI)^119,120^. Stretchable circuits and functional electronics can interface with various parts of the human body and collect signals with sufficient quality, resolution, and scalability^121^. Several high-quality papers have explained in detail the different approaches that have been used, which can be broadly divided into two main strategies: (1) Structural Engineering Approaches (2) Material-based Approaches. While these approaches enable varying degrees of stretchability, they often involve trade-offs between electrical conductivity, mechanical robustness, and fabrication complexity^122^.

### Structural engineering approaches

#### Buckling/wrinkling engineering

Buckling engineering imparts stretchability to rigid electronic materials by transforming global in-plane stretching into localized out-of-plane deformation, minimizing mechanical strain within the active device layers^123^. Buckling engineering enables the introduction of stretchability into otherwise non-stretchable functional layers, facilitating their integration into stretchable devices^124^. The buckling engineering method can be easily implemented by using a prestretched elastomeric substrate and subsequently releasing it^125^, which produces wavy structures^124^, or by using the molding method, and can be applied to a number of materials^126,127^. The primary limitation of buckling engineering is that electronic materials remain intrinsically stiff and brittle, with limited stretchability. In addition, there were some considerations about the large-scale buckling-formation process, further limiting its applications^128^.

#### Serpentine interconnect engineering and kirigami/origami engineering

In engineering, stretchable transistors and circuits utilize spatially heterogeneous layouts that include distinct device regions and separate areas for conductive interconnections (conductive traces). Conductive traces must exhibit high resilience to mechanical strain and are therefore fabricated on flexible substrates composed of soft materials. Stretchable traces have been achieved through various geometric designs, including serpentine patterns^129–131^, longitudinal wave structures^124,132^, and cut-based approaches^133^.

### Material-based approaches

Material-based approaches include the use of conductive polymers^134,135^, percolation filler networks^136^, and liquid metals^137^. However, these systems often exhibit non-uniform strain distributions due to their mechanically heterogeneous structure, leading to significant stress concentrations at interfaces between soft and stiff regions. Although geometry-based strategies such as serpentine, buckled and kirigami interconnections enable the use of high-performance rigid conductors in stretchable electronics, they have constraints such as limited stretchability compared to material-based approaches^138^, typically stretchability of serpentine  ~ 30–100%^139–141^, longitudinal wave ~50–200% and higher kirigami, (pattern-dependent) ~500–1000%^142,143^. In addition, strategies were adopted to develop flexible/stretchable electronic materials, such as conductive polymeric systems. Conductive polymer systems for stretchable circuits are generally categorized into conductive polymer composites (CPCs) and intrinsically stretchable conductive polymers (ISCPs).

#### Conductive polymer composites and intrinsically stretchable conductive polymers

ISCPs are engineered to be conductive and elastic at the molecular level^144^. Common design strategies for ISCPs include copolymerization, side-chain engineering, and dynamic bonding mechanisms such as hydrogen bonding, ionic interactions, or reversible cross-links. In CPCs, synthetic rubbers such as TPU and PDMS are used as the elastomeric matrix, while conductive fillers such as CNTs, graphene, silver nanowires, or metal nanoparticles are incorporated into the matrix^31,135,145,146^. Recent advances in soft bioelectronics highlight a shift from material-centric approaches to system-level mechanical design, in which functional layers are mechanically decoupled from soft biological substrates through compliant interlayers^147^.

#### Hybrid soft-rigid interconnection engineering

Marques et al. introduced EGaIn-based vertical interconnect accesses (VIAs) for reliable interfacing between liquid-metal circuits and rigid microelectronics^148^. The multilayer hybrid system exhibited stretchabilities of approximately 80% and maintained stable electrical performance over 1000 cycles at 40% strain, demonstrating the potential of VIA-based architectures for scalable stretchable electronics.

Lopes et al. introduced a reversible polymer-gel (Pol-Gel) transition strategy for the fabrication of ultra-stretchable chip-integrated circuits^149^. The approach enables self-soldering, self-encapsulation, and self-healing of stretchable electronics by exploiting solvent-induced phase transitions in styrene-isoprene-styrene (SIS) block copolymers. This method achieved strain tolerances exceeding 500% for chip-integrated circuits and approximately 1200% for printed interconnects, while maintaining stable electrical performance over 1000 stretching cycles at 100% strain. The proposed strategy significantly simplifies the integration of rigid microchips with soft electronics and represents an important advance toward scalable manufacturing of stretchable electronic systems.

A major challenge in stretchable electronics is the reliable integration of rigid electronic components, such as microcontrollers, LEDs, and resistors, with highly deformable interconnects. To address this issue, Tang et al. developed a mechanical interlocking strategy based on porous electronic vests (e-vests) and thermal bonding to create robust soft-rigid connections^150^. By combining metal-polymer conductors (MPCs) printed on TPU substrates with porous polyimide/copper connectors, the authors achieved soft-rigid interfaces capable of withstanding strains of up to 700%. Furthermore, the stretchable display maintained stable performance over 2000 stretching cycles at 100% strain, demonstrating the effectiveness of mechanical interlocking in enhancing the durability and reliability of stretchable electronic systems.

Although the aforementioned approaches aim to accommodate mechanical deformation, each presents specific implementation challenges. Buckling engineering enables the use of high-performance conventional electronic materials but often requires pre-strained substrates and complex architectures. Stiffness engineering mitigates mechanical mismatch and improves device durability, but may increase fabrication complexity. In contrast, intrinsically stretchable materials provide uniform deformability and conformability, making them attractive for wearable systems, although trade-offs in conductivity, carrier mobility, or long-term stability may arise.

Table 4 summarizes stretchable electronic systems based on different engineering strategies.

**Table 4:** Comparison of stretchable electronic systems based on different engineering strategies

Ref.	Strategy	Active material	Supporting materials	Mechanical properties	Electrical properties	Max strain (%)	Cycling stability
Buckling/Wrinkling engineering							
^124^	Buckling	Single-crystal Si	PDMS substrate, Al contacts	130 GPa (Si), 2 MPa (PDMS)	Semiconductor, Al: 3.5 × 10^7^ S m^−1^	30	100 cycles
^324^	Buckling	GaAs ribbons	PDMS substrate, AuGe/Ni/Au and Cr/Au contacts	85.5 GPa (GaAs)	Semiconductor	>20	Reversible stretching/compression
^325^	Buckling	Si nanoribbons	PDMS substrate, Cr/Au contacts	130 GPa (Si)	Semiconductor	~10	30 cycles
^126^	Wrinkling	Graphene/SWCNT	PI/PDMS substrate, Al_2_O_3_ dielectric	N/A	Conductive network	20	1000 cycles
Serpentine interconnect engineering							
^141^	Serpentine	Cu interconnects	Silicone elastomer substrate, Al pads	119 GPa (Cu)	5.9 × 10^7^ S m^−1^	90	N/A
^129^	Serpentine	TiW/Cu interconnects	Parylene-C substrate	119 GPa (Cu)	5.9 × 10^7^ S m^−1^	350	100 cycles
Kirigami/origami engineering							
^326^	Kirigami	CNT/PVA composite	Paper/Parylene support	N/A	Conductive CNT network	290	1000 cycles
^49^	Origami/Kirigami	Inorganic LEDs	PI substrate, Cu conductive layer	2.5 GPa (PI), 119 GPa (Cu)	Semiconductor, Cu: 5.9 × 10^7^ S m^−1^	20	N/A
Conductive polymer composites/intrinsically stretchable conductive polymers							
^135^	Conductive Polymer	PEDOT & STEC additives	Intrinsic polymer matrix, stretchable PEDOT conductor	N/A	3100–4100 S cm^−1^, > 100 S cm^−1^ at 600% strain	800	1000 cycles at 100% strain
^327^	Conductive Polymer	PEDOT	NaClO_4_ ductilization agent, PEDOT conductive network	42.9 MPa	5.38 S cm^−1^, strain-insensitive resistance	53.2	N/A
Hybrid soft–rigid interconnection engineering							
^328^	Soft–Rigid Hybrid	Serpentine Cu traces	Ecoflex substrate, soft microfluidic silicone enclosure	~ 100 GPa (Cu), ~180 kPa (Ecoflex)	5.9 × 10^7^ S m^−1^	N/R	N/R
^148^	Soft–Rigid Hybrid	EGaIn stretchable interconnect	PDMS substrate, FPCB (PI/Cu) with VIAs	Liquid metal, 2.28 GPa (PI)	3.4 × 10^6^ S m^−1^ (EGaIn), ~5.9 × 10^7^ S m^−1^ (Cu)	81.1	1000 cycles at 40% strain
^149^	Soft–Rigid Hybrid	Ag–EGaIn–SIS conductive composite	SIS substrate, integrated SMD microchips	~ 1 MPa (SIS), > 100 GPa (SMD components)	8.21 × 10^5^ S m^−1^	> 500	1000 cycles at 100% strain
^329^	Soft–Rigid Hybrid	ULPC (EGaIn–polymer composite)	TPU substrate, Si chips, SMD components	0.4–34 MPa (ULPC), 10–50 MPa (TPU), 130 GPa (Si)	2.5 × 10^5^ S m^−1^	> 600	10,000 cycles at 50% strain, 2500 cycles at 100% strain
^150^	Soft–Rigid Hybrid	Metal–polymer conductor (MPC)	TPU substrate, porous PI/Cu connector	3.3 MPa (TPU), 2.28 GPa (PI)	3.8 × 10^5^ S m^−1^ (MPC), ~ 5.9 × 10^7 ^S m^−1^ (Cu)	700	2000 cycles at 100
Liquid metal-based interconnections							
^330^	Liquid Metal	EGaIn liquid metal particles (LMPs)	PDMS/VHB substrate	Liquid, 2 MPa (PDMS)	2.4 × 10^6^ S m^−1^	> 1200	1000 cycles at 100% strain

N/A: Not reported in the original study.

*Si* Silicon, *PDMS* polydimethylsiloxane, *Al* aluminum, *GaAs* gallium arsenide, *Au* gold, *Ge* germanium, *Ni* nickel, *Cr* chromium, *PI* polyimide, *Al*_*2*_*O*_*3*_ aluminum oxide (Alumina), *Cu* copper, *Ti* titanium, *PVA* poly(vinyl alcohol), *LED* light-emitting diode, *PEDOT* poly(3,4-ethylenedioxythiophene), *STEC* stretchable thermoelectric composite, *NaClO*_4_ sodium perchlorate, *Ecoflex* silicone elastomer, *EGaIn* eutectic gallium-indium, *FPCB* flexible printed circuit board, *VIA* vertical interconnect access, *SIS* styrene–isoprene block copolymer, *SMD* surface-mount device, *ULPC* ultra-low-percolation composite, *TPU* thermoplastic polyurethane, *MPC* metal-polymer conductor, *VHB* very high bond (acrylic foam tape).

## Wireless communication through textile solutions

The Internet of Things (IoT) implication allows sensors data to connect to networks and provide real-time analytics for personalized insights^151,152^, in contrast to traditional systems that often require rigid construction and wired interfaces to external data acquisition hardware^153^. Therefore, wireless communication is essential as it enables continuous monitoring of detected signals without restricting users, allowing comfortable, irritation-free measurements. Such systems include Bluetooth, Wi-Fi, and NFC, with Bluetooth and Wi-Fi supporting comparatively longer-range communication, whereas NFC (near-field communication) is optimized for short-range, low-power interactions^154^. Wireless communication in wearable textiles integrates sensors and circuitry directly into fabrics, employing technologies such as NFC, Bluetooth, and WiFi to enable seamless data transfer for health monitoring and interactive feedback. NFC-based systems can enable short-range wireless data transmission and, in passive configurations, battery-free operation through inductive coupling^155^. A major limitation of NFC technology is its short operating range, as passive NFC devices typically need to remain within the near field of the reader. Passive inductor–capacitor (LC) circuits can be embedded in fabrics to detect physical changes, such as strain, and wirelessly transmit the resulting signals through inductive coupling to a nearby reader. Therefore, this approach goes beyond rigid devices to create seamless integrated and comfortable e-textiles^156,157^. In this context, wireless device technologies that can non-invasively interface with relevant regions for continuous monitoring are of great interest^158^. This approach uses a network of sensors embedded in the garment to continuously measure human physiological signals, which is essential for reliable long-term monitoring. In diverse application scenarios, including vital signs monitoring and fitness tracking, simultaneous data collection from multiple anatomical locations can further improve the utility and reliability of these measurement systems^159^.

This wireless communication can be achieved using a chip-based NFC system coupled to a coil antenna, which can receive power and transmit data to a nearby reader^160–163^. A textile antenna can transmit measured data via NFC, Bluetooth, or Wi-Fi, provided that it is designed and tuned for the corresponding frequency band and connected to the appropriate communication module. Depending on the system architecture, power may be supplied by an integrated power source or through wireless power-transfer mechanisms such as inductive coupling^164,165^.

The integration of antenna systems into garments represents a promising area of research, as textile-based antennas offer flexibility and elasticity, enabling them to conform to the human body’s shape while maintaining wearer comfort. Textile antennas can provide wireless communication functionality while maintaining the flexibility and conformability required for garment integration. In some designs, the antenna itself can additionally serve as a sensing element^166^. For instance, a multi-node, battery-free wireless sensor network has been demonstrated for spinal-posture monitoring using a smartphone as the reader^167^. There are many other factors that must be taken into account when designing antennas, such as homogeneity, high conductivity, flexibility, and elasticity^168^. For wearable applications, textile antennas should ideally exhibit low weight, flexibility, mechanical robustness, and washability^169,170^. Textile antennas can be fabricated using several techniques, including weaving, knitting, embroidery, and printing with conductive inks, each of which presents specific advantages and limitations^171^. A comprehensive understanding of textile material characteristics is essential to mitigate parasitic effects on antenna performance. Once the fabric adapts to the body’s surface, its structure may bend or deform, potentially affecting antenna performance. The dielectric properties of the textile substrate influence antenna characteristics such as impedance matching, resonance frequency, radiation efficiency, and bandwidth^169,172,173^. In addition, factors such as mechanical strength, fatigue tolerance, and the electromagnetic properties of textile materials, including permittivity and loss tangent, are considered^168^. The moisture content of the fabric can alter its properties, including its mechanical rigidity and permittivity. Moreover, because wearable antennas operate in close proximity to the human body, the high permittivity and dielectric losses of biological tissues can significantly influence antenna performance^174^. Accordingly, the electromagnetic exposure associated with wearable antennas should be evaluated to ensure compliance with applicable safety requirements^168^. Triboelectric nanogenerator (TENG)-based direct sensory transmission (TDST) was introduced, enabling the conversion of triboelectric output into stable wireless signals in the frequency domain through LC resonant, thus eliminating the need for additional electronic components^175^. Unlike previous self-powered systems that require energy storage or resonant modulation, this approach relies on discharge-induced ultrafast transients to achieve direct wireless transmission. Later, they enabled much longer transmission distance by replacing coil-based near-field frequency-modulated communication with direct electromagnetic radiation generated by ultrafast discharge events (SWISE)^176^. In this paper, mechanical motion is converted into a fast discharge event that inherently radiates a detectable wireless electromagnetic signal, enabling battery-free long-distance sensing without electronic transmitters.

Electronic components such as modules for signal generation, power supply and management, signal modulation, and transmission are often rigid and bulky, limiting their application in wearable systems. Although textile antennas enable flexible and garment-integrated radiating elements, their operation still requires electrical input from communication modules or integrated circuits. However, recent advances in materials science have enabled the creation of flexible, stretchable, and lightweight energy storage and harvesting solutions. Integration of energy storage and harvesting technologies is essential for developing self-sustaining systems that minimize reliance on external power sources and improve device longevity. These integrated systems ensure the continuous operation of vital sensors and processors for real-time health monitoring. Portable and wearable self-powered systems are gradually replacing bulky electronic devices.

Therefore, numerous efforts have been made to develop chip-free, self-powered systems. This dependence has motivated research into wireless- and self-powered wearable networks that integrate energy generation and communication directly into textile structures. This approach involves the triboelectric nanogenerator (TENG) based on principles of triboelectricity (a phenomenon of contact-induced electrification) and electrostatic induction, which converts mechanical energy from the human body into electrical energy^177,178^. Numerous TENG-based self-powered sensing devices have been developed, demonstrating the significant potential of TENG as a battery-free sensor system and a promising solution for achieving self-powered wireless body area networks. Integrating TENGs with wireless transmission components can enable self-powered operation, potentially improving portability and long-term usability^179^. Recent studies have shown that piezoresistive sensing can be integrated with other transduction mechanisms, such as triboelectric sensing, to expand sensing modalities, including non-contact detection, while preserving piezoresistive capabilities^180^. They demonstrated a practical architecture where a TENG can wirelessly deliver power efficiently at short range, by converting pulses into a resonant sinusoid, and send sensor information much farther than a chipless self-powered wireless sensor by encoding information in signal amplitude and resonant frequency, without batteries or active electronics. However, TENG-based wearable wireless sensing systems that rely on signal amplitude as the sensing parameter have some limitations. The amplitude in that system can be strongly affected by distance and orientation and can be highly unstable due to environmental factors such as humidity. In addition, connections between sensor nodes on the human body interfere with physical activities, and wireless technologies require each sensor node to be powered separately for long-term operation.

Although early self-powered wireless transmission strategies such as TDST^175^ and SWISE^176^ established frequency-based and discharge-induced signal emission, respectively, more recent efforts such as CSDC-TENG WBAN^181^ have leveraged those principles in a fully wearable, multi-node body area network context. Signals from different body sensors are collected, identified by their frequency, and sent to a computer or phone for real-time motion monitoring^181^. This approach enables multi-joint movement monitoring using a self-powered wireless body area network (SWBAN)^181^.

Single-function electronic devices are increasingly insufficient to meet the functional requirements of portable electronics in the intelligent era. Powering wearable sensors requires compact batteries or energy harvesting from ambient sources, particularly because wireless data transmission and high sampling rates can increase energy consumption. Building upon this concept, recent fiber-based wireless body area networks further integrate triboelectric energy generation, sensing, and near-field wireless transmission directly into textile fibers, achieving fully wearable, chip-free, and washable systems. Together, these studies (TDST^175^ and SWISE^176^) illustrate the progression of self-powered wireless sensing toward frequency-domain and direct electromagnetic signal transmission. The self-powered, chip-free textile WBAN developed by Wei et al.^182^ represents a hardware-level approach to textile-based WBANs by integrating self-powered sensing and wireless interaction directly into fibers. Therefore, the aforementioned triboelectric–electromagnetic induction coupling mechanism harvests mechanical energy generated by human-body movement and converts it into electrical energy. This low-frequency electric signal (1 Hz) can then be transformed into a high-frequency electromagnetic signal for wireless transmission^182^, as shown in Table 5.

**Table 5:** Wireless communication through Textile Solutions

Study	Wireless transmission method	Power supply	Flexibility of components	System scale	Ref.
NFC-based inductive coupling with passive textile relays	NFC-based inductive coupling	Powered by NFC reader	Soft and textile-based	Body-scale network	^167^
Wireless Communication and Energy Harvesting					
Stretchable passive RFID bodyNET	Passive RFID / inductive coupling (13.56 MHz)	Battery-free passive tag; powered/read by RFID initiator	Stretchable on-skin tag; flexible textile-mounted readout	Body-area sensor network	^331^
Magnetic-resonance-coupled TENG wireless system	Magnetic resonance coupling (resonant inductive coupling)	Self-powered	Semi-flexible	Device/patch-level	^180^
TDST-based SS-WSN	Near-field inductive coupling (frequency-shift)	Self-powered	Flexible textile	Patch, textile-pixel	^175^
CSDC-TENG WBAN (motion monitoring)	Near-field inductive coupling	Self-powered	Semi-flexible	Body-area network (multiple sensing nodes)	^181^
SWISE, Discharge-induced displacement current	Discharge-induced displacement current (EM radiation)	Self-powered	Soft & Flexible	E-sticker	^176^
Fiber-WBAN	Near-field electromagnetic coupling (LC resonance)	Self-powered	Fully soft, fiber-based	Fiber-level (textile-integrated)	^182^

*BLE* bluetooth low energy, *NFC* near field communication, *TENG* triboelectric nanogenerator, *TDST* triboelectric-driven smart textile, *SS-WSN* self-sustainable wireless sensor network, *CSDC* contact-separation direct current, *WBAN* wireless body area network, *SWISE* self-powered wireless intelligent sensing electronics, *EM* electromagnetic, *LC* inductor-capacitor.

## Signal processing, multimodal fusion, AI integration

Wearable textile electronics represent an emerging area of research for personalized medicine applications^9^. As digital health advances rapidly, wearables can first detect abnormal patterns and then transform them into digital biomarkers, ultimately serving as valuable tools for diagnosing and monitoring diseases and conditions. Continually tracking metrics such as physical activity^10,183^, heart rate^184,185^, and sleep patterns^186–188^, portable devices can help identify subtle changes that precede clinical symptoms^189,190^. In addition, for people with diabetes or hypertension, these devices provide continuous data that help monitor condition stability and treatment effectiveness^191^, and have been reported to produce more precise and reliable predictions than clinical laboratory measurements^192^.

### Signal processing

Signal processing is fundamental to the effectiveness of wearable devices because raw signals collected under real-world conditions are frequently affected by motion artifacts, environmental noise, and variability at the sensor-skin interface^193^. Robust preprocessing pipelines, which generally include data cleansing, normalization, filtering, artifact suppression, and signal quality assessment, are essential for extracting meaningful physiological parameters^194^. Traditional digital signal processing methods, such as band-pass filtering, spectral analysis, and time-frequency transforms, have been widely implemented. More recently, data-driven approaches have gained prominence, including ML models that enable automatic feature extraction and adaptive noise reduction. DL architectures, such as convolutional neural networks (CNNs) and recurrent neural networks (RNNs), have demonstrated particular promise in managing non-stationary biosignals and capturing complex temporal dependencies. Future signal processing for wearable devices is expected to advance toward multimodal fusion^195,196^, self-supervised learning^197^, and energy-efficient edge processing^198,199^, thus supporting more robust, personalized, and clinically reliable health monitoring in unconstrained environments.

However, transforming data into digital biomarkers poses significant computational challenges, and there is a lack of standardized methods and validation protocols for their discovery^200^. Most wearable devices process raw sensor data locally and typically utilize device-specific methods, often by using highly specific functions to process, clean, normalize, and compute features. This leads to a lack of uniformity and reproducibility, limiting scalability, and impeding cross-technology validation^201^. Therefore, a significant challenge in data analysis is the lack of standardized protocols for data collection, processing, and analysis^202,203^. The lack of standardized methodologies impedes the reproducibility of research findings and complicates the validation of device performance in diverse populations and real-world settings^202,204^. The establishment of standardized guidelines and frameworks for data processing is urgently required. For example, researchers have developed tools to generate physiological features such as Feature generation tooLkIt for weaRable daTa (FLIRT), although these tools may remain signal-specific^205^. Moreover, the use of open-source platforms and repositories for pre-trained models, annotated datasets, and analytical pipelines can enhance research outcomes. Providing standardized guidance on data pre-processing, feature extraction, and analytical methods facilitates more reliable and comparable results, advances personalized health and precision, and supports the development of this emerging field^206^. Some researchers and developers emphasize the importance of accessing raw unfiltered data and comprehensive metadata to improve transparency and allow consistent preprocessing and analysis methods^207^. Therefore, effective collaboration among industry stakeholders, academic institutions, and regulatory authorities is essential to ensure that technological developments adhere to established standards^208,209^.

### Multi-modal fusion

Advancing health care outcomes requires integrating multiple data streams rather than relying on individual measures^210,211^. In that direction, multi-nodal designs have emerged as a significant innovation in wearable systems, enabling the detection and processing of multiple types of stimuli from a single sensor^212–214^. By integrating multiple sensing mechanisms, these sensors provide richer, higher-fidelity data than single-function sensors, enabling more comprehensive assessments in fields such as health monitoring and HMI^215^. The integration of multi-modal fusion techniques offers a framework for a more comprehensive understanding of health monitoring that extends beyond clinical settings^216–218^. This cohesive representation would improve the accuracy and robustness of the systems^219,220^.

In wearable devices, multimodal fusion integrates data from multiple sensors to provide a more accurate, reliable, and comprehensive assessment of user health and activity than any single sensor can achieve^221–223^. Integrating raw data from multiple sources would improve the accuracy and reliability of health assessments. For example, in human-computer interaction, multimodal data fusion integrates information from various modalities, such as eye gaze, facial expressions, and motor skills, thus enhancing understanding of complex interactions between behavioral and neurological factors^224,225^. In healthcare informatics and biomedical research, multimodal data fusion combines multiple biomarkers to improve diagnosis, treatment planning, and personalized medicine^226,227^. High-performance flexible sensors have broad application prospects in various fields, including robotics, skin, and wearable devices. A single sensing type is constrained by its own limitations and cannot adapt to different applications. There have been cases of dual-mode flexible sensors based on the combination of piezoresistive and triboelectric sensing^228,229^. For instance, in the era of big data and AI technology, electronic devices are no longer limited to a single sensing or actuation function. In the future, wearable electronic devices with powerful data processing capabilities will become a new interface of HMI.

Smart healthcare systems and the Internet of Medical Things (IoMT) are expected to utilize multimodal data fusion to enable real-time monitoring, predictive and preventive healthcare, and personalized treatment strategies^230,231^. However, implementing data fusion in healthcare applications requires addressing several factors. The selection of the sensor should be one of the primary factors that must be carefully considered, depending on the target outcome^232^. Nevertheless, challenges remain in real-time data collection^233,234^, noise reduction and filtering (such as adaptive filtering, AI/DL, and frequency-domain methods)^235–237^, time synchronization^238^, and normalization of diverse data streams to ensure comparability. Especially in healthcare applications, calibration is critical due to environmental factors, electromagnetic interference, and electronic variations^239^. Moreover, careful calibration would help mitigate errors and deviations and ensure that sensor readings align with known standards and reference measurements^240,241^. However, there are potential challenges, including separating overlapping signals from different stimuli^242^. For example, strain sensors with integrated temperature-insensitive elements can obtain signals for the body surface while monitoring activity data^243,244^. This could improve the precision of next-generation sensors by enabling the detection of subtle changes that a single sensor cannot detect, improving accuracy and enabling diverse applications, leading to more versatile and adaptable devices in the future^245,246^.

### AI integration

The vast amount of data collected by these sensors can be analyzed using AI, including ML and DL algorithms, to identify complex patterns, extract meaningful features, and provide valuable health insights^247–249^. AI can further enhance these capabilities by enabling adaptive responses during real-time data analysis^250,251^. For instance, in wearable and sensor-based systems, AI models can adapt to an individual’s unique physiological baseline, behavioral patterns, and environmental context, enabling tailored feedback over time^252^. These systems continuously update internal representations based on longitudinal data, refining feedback to reflect personal trends rather than relying on generic norms. AI also enables personalized feedback by learning individual-specific patterns rather than relying solely on population-averaged thresholds^253,254^. This personalization can increase the relevance and interpretability of feedback and improve user engagement, particularly in long-term monitoring scenarios^192,255,256^. Wearable devices also enable remote, continuous data collection, allowing patients to capture physiological measurements outside clinical settings and over extended periods^253^. Furthermore, the seamless integration of sensor data with smartphone applications supports user-friendly monitoring^257^. Integration with advanced AI methods can further enhance these capabilities by enabling precise motion tracking, multimodal signal processing, and dynamic feedback, thereby supporting real-time healthcare applications.

Although multimodal sensors generate substantial volumes of data, conventional computing architectures continue to rely primarily on centralized processing^227,258–260^. Integrating sensing, memory, and computation at or near the sensor can enhance energy efficiency, responsiveness, and scalability, while more closely emulating the efficiency observed in biological sensory systems^261^. Specifically, the advancement of modern wearable systems necessitates rapid information processing and analysis, as well as substantial data-storage capacity^262^. AI frameworks can combine deep learning models, particularly convolutional neural networks, with multisensor information-fusion methods to improve accuracy, robustness, and effectiveness^261^. AI systems also adapt to various types of noise and signal variations, thereby enhancing analytical accuracy, reducing noise, and enabling real-time decision-making^263,264^. Future AI systems, especially those incorporating multiple sensors such as wearables and IoT platforms, should prioritize edge intelligence over centralized computing^265,266^. In parallel, ethical challenges regarding data protection remain and may affect the adoption of AI-driven healthcare solutions.

Soft, wearable sensors have initiated a remarkable technological revolution, enabling the development of more intelligent platforms that exchange information between humans and their environment^267^. The seamless human-AI interaction will create a continuous loop between users and AI, allowing real-time monitoring, transforming the health care system, and opening a new area of personalized health monitoring to a more personalized model^268–270^.

Challenges associated with the integration of AI include privacy concerns^271–275^, data ownership issues^276–279^, and broader ethical concerns related to the generation, collection, storage, and analysis of private data. These concerns are closely associated with issues of confidentiality, integrity, responsibility, authenticity, and secure data management^280^. The development of experimental tools will certainly generate new information, but at the same time, massive amounts of data will have to be properly processed, analyzed, and interpreted. This poses several technological challenges in terms of interpreting the data collected, which could be addressed using generative AI^281–283^. As AI-assisted wearable systems become increasingly integrated into healthcare decision-making, regulatory frameworks such as the European Union Artificial Intelligence Act (EU AI Act) may influence requirements related to transparency, risk management, and system validation.

Current regulatory frameworks for AI-integrated textiles combine general AI and data protection laws, such as the EU AI Act and the General Data Protection Regulation (GDPR), with principles focused on human rights and safety^284,285^. For example, the EU AI Act adopts a risk-based approach for AI systems, categorizing them by risk level and imposing stricter regulations on high-risk applications^286^. Such regulations can affect wearable-system design, AI deployment, validation, explainability, and data handling. In parallel, GDPR and related data protection laws govern the collection and use of personal data, making transparency, secure processing, and an appropriate lawful basis for data processing essential for AI-powered smart textiles^287^. Compliance with these frameworks may require appropriate data-protection measures, including secure data processing and, where applicable, anonymization or encryption to reduce the risk of unauthorized access and misuse. At the organizational level, comprehensive guidelines are being developed to align with internationally recognized AI frameworks such as those from the European Union (EU), Organization for Economic Co-operation and Development (OECD) and Singapore’s Model AI Governance Framework^288–290^.

For wearable healthcare technologies, compliance with frameworks such as the EU AI Act and Software-as-a-Medical Device (SaMD) pathways may introduce additional validation, documentation and post-market monitoring requirements, potentially slowing large-scale deployment while improving clinical reliability, transparency, and patient protection. These regulatory frameworks increasingly require developers to clearly define intended medical use, characterize input physiological data, demonstrate reproducible algorithm performance, and establish risk-management and post-deployment monitoring strategies. Region-specific initiatives, including Singapore’s AI in Healthcare Guidelines (AIHGI) and the SaMD regulatory framework, further emphasize responsible AI deployment, data governance, algorithm transparency, and human oversight in AI-assisted healthcare systems. The key regulatory frameworks relevant to AI-integrated wearable and smart textile systems are summarized in Table 6.

**Table 6:** Regulatory frameworks and their design implications for AI-integrated wearable textile systems

Regulatory framework	Key focus	Design implications for wearables
GDPR	Privacy and data governance	Edge processing, encrypted transmission, and data minimization
EU AI Act	Transparent and trustworthy AI	Explainable AI, human oversight, and lightweight inference
SaMD	Clinical safety, effectiveness, and validation	Calibration stability, reproducibility, and multimodal validation

*GDPR* general data protection regulation, *EU AI Act* European Union Artificial Intelligence Act, *SaMD* software as a medical device.

Despite these developments, the emergence of AI-integrated e-textiles—textiles with embedded electronics and AI functionality—creates new demands. Currently, regulatory approval protocols are often time-consuming and are not fully adapted to the unique characteristics of wearables integrated with AI. As a result, streamlining these regulatory pathways may facilitate the transition of electronic textiles from research laboratories to real-world applications. Future research should also focus on standardization protocols and the integration of robust signal-processing and AI-assisted algorithms. Therefore, it is more urgent than ever to consider privacy and cybersecurity factors.

## Challenges and future perspectives

Despite rapid progress in flexible and stretchable wearable sensing technologies, most reported systems remain limited to proof-of-concept demonstrations under controlled laboratory conditions. Although these studies present promising technologies, there is insufficient evidence to determine whether these systems are truly ready for practical use. Comprehensive validation remains lacking, raising concerns about potential issues with long-term wear, sweat, washing, real-world calibration, reproducibility, fabrication costs, scalability, and readiness for real-world settings. Furthermore, sensing accuracy and long-term device reliability remain challenged by calibration drift, inter-user physiological variability, motion artifacts, changing environmental conditions, and washability requirements associated with wearable applications.

Therefore, practical translation still requires stronger evidence of device-to-device uniformity, long-term wearability, and real-world validation. Validation should also include benchmarking against established devices and performance evaluation under real-world and daily-life conditions. Future wearable studies should emphasize participant-based validation to assess performance under realistic conditions, including repeatability and inter-subject consistency. In that direction, validation across diverse patient populations should be strongly encouraged^291^.

Beyond reliability, the widespread deployment of wearable technologies also introduces important privacy, ethical, and regulatory challenges. As fabrics become capable of continuously sensing, memorizing, and interpreting human activity, these capabilities raise concerns regarding biometric surveillance, ownership of physiological data, military applications, and behavioral profiling. As wearable healthcare technologies continue to advance, cybersecurity becomes increasingly critical due to the continuous acquisition and wireless transmission of sensitive data. Large volumes of sensitive biometric data require protection from unauthorized access, data breaches, and cyberattacks through secure systems and appropriate data-management practices. Consequently, increasing emphasis should be placed on secure wireless communication, encrypted data transmission, and edge-based processing strategies that reduce the reliance on cloud-dependent architectures. Researchers must implement robust data security measures, ensure data integrity, acknowledge potential biases, and establish clear accountability for data handling^292,293^. In wearable research, robust data-management frameworks and ethical standards are essential, including those outlined in the GDPR, which increasingly require researchers and institutions to collect only the minimum necessary information for specified purposes and to retain data solely for the duration needed to achieve the original research objectives^294^. A major ethical challenge in research is to minimize risk while ensuring meaningful scientific results. Therefore, researchers should ensure that they recruit a scientifically valid number of participants, based on the research questions and the number needed for each study, while minimizing unnecessary participant involvement, risks, and experimental burden^295,296^. The purpose of the study must be considered: if the research does not aim to support statistical generalizations, a small sample size may be sufficient^297^. Moreover, including too many participants can prolong the trial and delay the scientific outcomes^295^.

Equally important is the use of appropriate encryption to prevent unauthorized access and data breaches, which could compromise participants’ privacy and confidentiality^298,299^. Therefore, robust data-protection measures should be implemented to ensure privacy safeguards, such as end-to-end encryption and secure data storage protocols^300^. Another commonly used practice that researchers can adopt is promoting transparency. This can be done by incorporating open-access (OA) data, which makes this information more freely available, fosters innovation and collaboration between institutions, and ultimately builds trust and enables informed decisions through initiatives such as open data portals and FAIR (Findable, Accessible, Interoperable, and Reusable) data principles^301–303^. Strict anonymization procedures should be established to safeguard against potential re-identification and preserve public values^304^, while ensuring that such measures do not unnecessarily restrict data access and that appropriate safeguards are in place to prevent deanonymization^305^. When making data openly accessible, addressing privacy concerns extends beyond legal compliance; it is essential to uphold and protect the rights and autonomy of individuals who provide their data to wearable technologies^306,307^. Furthermore, a high level of transparency should be guaranteed not only in how data will be collected but also in how it is used, stored, and managed^308,309^. Ethical data-collection practices require transparent informed-consent procedures that address study objectives and potential risks, and the secure management of personal biometric data, while ensuring that participation is voluntary and that individual autonomy and decision-making are respected^310^. Therefore, researchers developing textile-based wearable healthcare systems must consider not only sensing performance but also privacy protection, secure data transmission, and ethical data management strategies.

In conclusion, the increasing adoption of real-time wearable sensing platforms opens new doors, but significant challenges remain for the secure management of personal health data. Collectively, ethical conduct, privacy protection, and robust security measures are essential to maintain public trust in scientific research and to ensure that technological advances are implemented responsibly while minimizing potential adverse consequences.

## Outlook

A key finding of this review is that the development of textile-based sensors is currently characterized by important trade-offs between sensing performance and practical implementation. While some sensor architectures achieve high sensitivity, they may exhibit limitations in stretchability, durability, large-scale manufacturability, or seamless textile integration. Conversely, approaches that are highly compatible with textile manufacturing processes may exhibit limitations in sensing performance or device functionality. The comparative analysis highlights that no single solution currently optimizes all evaluation criteria simultaneously. Therefore, future research should focus not only on improving individual performance metrics but also on achieving a more balanced integration of sensitivity, stretchability, durability, manufacturability, and textile compatibility to enable scalable and application-ready wearable sensing systems.

Moving forward, future research should prioritize refining sensor accuracy and reliability, developing standardized metrics for health assessment, and employing AI-driven analytics, while upholding ethical standards of scientific research. The revolution in wearables towards multimodal wearable devices is providing solutions that seamlessly bridge the physical and digital worlds, enabling continuous health monitoring. These efforts may contribute to wearable technologies that can provide continuous real-time monitoring of individuals in their environments.

## Supplementary information

Supplementary Data

Transparent Peer Review file

## Data Availability

No new experimental data were generated. Data extracted from previously published studies were compiled and analyzed for the comparative assessment presented in the tables. A workbook provided at https://doi.org/10.6084/m9.figshare.33265395 contains the study-level data extracted from the papers, including sensitivity, stretchability, durability (number of cycles), textile compatibility, and ease of fabrication, along with the corresponding scores used in the comparative assessment. The second worksheet provides the predefined quantitative scoring thresholds and qualitative assessment criteria used to assign these scores.
