# Supplementary material for: From stretch﻿able materials and functional fibers to intelligent textile sensing systems
Source: Commun Mater. 2026 Oct 21;7(1):273. doi: 10.1038/s43246-026-01333-4 (PMC13686928; doi:10.1038/s43246-026-01333-4)
Supplement: Supplementary file 1 [file 43246_2026_1333_MOESM2_ESM.pdf]

nature portfolio

Peer Review File

From Stretchable Materials and Fibers to Intelligent Wearable Systems

Corresponding Author: Ms Maria Papaefstathiou

This manuscript has been previously reviewed at another journal. This document only contains information relating to versions considered at Communications Materials.

This file contains all reviewer reports in order by version, followed by all author rebuttals in order by version.

Version 0:

Reviewer comments:

Reviewer #1

(Remarks to the Author)
This manuscript presents a review of recent advances in wearable electronics, covering material innovations, mechanical design strategies, textile integration, wireless communication, and AI-enabled signal processing. The topic is timely and of interest, particularly given the increasing demand for soft, conformable, and garment-integrated sensing systems in healthcare and human–machine interfaces. Some comments are as follows:
1. The title emphasizes: “From Rigid to Flexible, Stretchable Fibers”, which suggests that the manuscript will focus on fiber-based electronics and textile-integrated sensing systems as the central theme. However, in its current form, only a relatively small portion of the manuscript (primarily Figure 3 and related discussion) is dedicated to fiber/yarn-based systems.
2. While the manuscript provides a wide coverage of the field, it currently reads largely as a descriptive compilation of literature rather than a critical and forward-looking review. More importantly, there is a significant mismatch between the stated focus on fiber-based wearable systems and the actual content of the manuscript.
3. The manuscript is organized into sections: materials, circuits, wireless, AI, but it lacks a coherent conceptual framework, which is not shown in Figure 1.
4. A major limitation of the manuscript is the absence of systematic quantitative benchmarking. Although individual values are occasionally mentioned (e.g., GF, strain limits).
5. Fiber electronics section lacks depth and novelty. For example: important missing topics such as thermally drawn multifunctional fibers.
6. The manuscript mentions healthcare applications and digital outcome measures. However, there is limited discussion on real-world deployment such clinical validation, calibration and reproducibility, etc.

Reviewer #2

(Remarks to the Author)
This paper provides a comprehensive review on the development of wearable electronics, focusing on the technological transition from rigid devices to flexible, stretchable fiber integrated systems. It systematically summarizes advanced materials, mechanical design strategies, stretchable conductive circuits, textile based wireless communication, multimodal signal fusion, and AI integration for digital health applications. The review also discusses current challenges including system integration, data security, privacy issues, and ethical concerns, as well as future directions for soft wearable technologies. The topic is highly relevant to flexible electronics and smart textiles, and the review covers sufficient scope with clear logic. However, several improvements in presentation, structure, readability, and technical description are needed to enhance its quality and academic impact.
1. Figures throughout the manuscript vary significantly in size, layout, and annotation style. All figures are too small to clearly show structural details, working principles, and fabrication flows.
2. The scale, font, and labeling of subfigures are inconsistent, which greatly reduces readability and visual coherence. All figures should be standardized, enlarged, and uniformly formatted.
3. The section “Material & Mechanical Innovations (From Rigid Systems to Stretchable Textiles via Fiber-Level Integration)” covers a wide range of content including conductive materials, percolation theory, crack engineering, auxetic structures,

breathability, fiber fabrication, and textile integration. All these topics are merged without clear subsections, making the logic difficult to follow. It is strongly suggested to divide this section into multiple clear subheadings. Other sections should also consider this division optimization.
4. Many paragraphs switch abruptly between materials, mechanisms, and applications without smooth connecting statements. Transitional sentences and clear topic guidance are needed.
5. The opening statement covers an overly broad range of wearable devices, while the manuscript mainly focuses on stretchable fiber-based composite devices and textile-integrated systems. The core theme, scope, and logical outline of the review are not clearly highlighted. The authors should rewrite and narrow the introduction to clearly state that this review concentrates on fiber-based stretchable wearable electronics, rather than general wearables.
6. Stretchable devices and stretchable circuits have inherent overlaps in their preparation methods. This article specifically discusses the content of stretchable circuits, but it has little relevance to the previously mentioned fiber-based stretchable devices for fabrics. Careful consideration is needed for classification, and it is necessary to optimize it carefully to ensure a more logical structure. The current structure is rather chaotic.

Reviewer #3

(Remarks to the Author)

Reviewer Comments — Recommendation: Major Revision (borderline reject)
The manuscript surveys the transition from rigid to fiber-based stretchable wearables, and the authors have clearly invested effort in the figures (Figure 2 on percolation/cracking/auxetic mechanics and Figure 3 on fiber manufacturing are pedagogically well-drawn) and in the regulatory section, which is a relatively uncommon addition to a hardware-focused review. With 356 references, the literature base is also broad. In its current form, however, the review does not establish a distinct contribution alongside an unusually crowded recent literature, and the scope mismatch with the title needs to be addressed before the manuscript can be evaluated on scientific merit.
The most fundamental concern is that the differentiator is not made. The last 24 months have produced multiple specialist reviews that cover each of the manuscript's sub-sections in substantially greater depth, none of which are positioned against in the introduction: Yin, Wang, Tat and Chen in Nat Rev Bioeng (2024) on motion artefact management for soft bioelectronics, integrating materials, device design, and algorithmic intervention; Xu, Xiao and Chen in Natl Sci Rev (2024) on stretchable fiber strain sensors for biomonitoring; Liu et al. in Nano-Micro Lett (2022) on functionalized fiber-based strain sensors; Chen, He, Du and Lee in Nano Energy (2023) on textile-based wearable electronics through the fiber–yarn–fabric hierarchy; Xu et al. in Nano-Micro Lett (2025) on smart textiles for personalized sports and healthcare; the recent system-on-fiber review in Adv Sci (2025); and Fang et al. in Adv Mater (2025) on ionic conductive textiles. The introduction must explicitly state what gap this review fills relative to those works. As written, an informed reader cannot answer "why this review, why now."
The title–scope mismatch is the second concern and is, in my view, the issue most likely to determine the manuscript's fate at Communications Materials. The title commits the review to fibers, but only roughly a third of the body actually concerns fiber-level engineering; the remainder spans thin-film stretchable conductors, stretchable circuits, wireless communication, signal processing/AI, and ethics. Each of these is a legitimate sub-field, but each is treated at tutorial depth, and as a result no single sub-section reaches the specialist-level contribution that Communications Materials readers expect. The authors should either (i) honestly narrow the scope and the title to fiber-level innovations and engage with the specialist literature above in real depth, or (ii) keep the broad scope but explicitly reposition the review around an integrating thesis that the existing surveys do not provide — for example, the systems-engineering challenges of going from a fiber to a garment-level closed loop. The current uncommitted middle position is the weakest of the three options.
Technical depth and quantitative anchoring are also insufficient for a 2026 review on this topic. The materials section repeatedly cites gauge factors and stretchability values from individual studies without consolidating them into a comparison table, so a reader cannot easily place a new device against the field. The stretchable-circuits section invokes the standard buckling/stiffness/intrinsic taxonomy without adding new analytical structure on top of it. The wireless and AI/signal-processing sections describe CNN, RNN, multimodal fusion, and edge intelligence at a level that does not engage with current state-space, model-architecture, or on-device-inference details. At least one summary table consolidating gauge factor, stretchability range, response time, washing-cycle stability, and integration method across the major fiber sensor families would substantially strengthen the review.
Two further points deserve mention. The author team's real strengths — wearable signal processing and digital biomarkers in the BMHT group at ETH, and physiological-signal AI from the Khalifa side — do not come through clearly in the manuscript, because the AI/signal-processing section reads as a generic survey rather than as an authored perspective. I would strongly encourage the authors to use the revision to bring their own programmatic viewpoint to the front, particularly in the signal-processing and digital-biomarker discussions; this would also be the most natural way to manufacture a real differentiator. The manuscript also needs sentence-level English editing throughout: there are recurrent issues such as "rigid electronics may delaminate due to displacement," "smart appear" (apparel), "battery offerings," "harware-level paradigm shoft," and several broken constructions. For a senior-authored review this level of polish is below what reviewers will expect.
Finally, the regulatory/ethics section, while uncommon for a hardware review and a potentially valuable differentiator, is currently treated at the same depth as the rest of the manuscript and reads more as a checklist than as an analysis. If the authors choose to keep this section, it would benefit from being explicitly framed as one of the review's distinguishing contributions, with concrete engagement with how GDPR, the EU AI Act, and SaMD pathways change design decisions for fiber-based wearables specifically.
In summary, without (i) an explicit gap statement against the 2024–2026 reviews above, (ii) resolution of the title–scope mismatch through either narrowing or repositioning, (iii) real technical depth and quantitative anchoring in at least the

materials and circuits sections, and (iv) sentence-level editing throughout, the manuscript overlaps too heavily with stronger recent reviews to justify publication in Communications Materials. With these revisions, however, the work could become a useful entry point to a difficult literature, particularly if the authors lean into their signal-processing and regulatory perspectives as the differentiating axes. I look forward to a revised version.

Version 1:

Reviewer comments:

Reviewer #1

(Remarks to the Author)
The authors have thoroughly addressed all of my comments and suggestions which I believe the revised manuscript is in a good shape to be published in this journal.

Reviewer #2

(Remarks to the Author)
The overall quality of this manuscript has been improved a lot. So i recommend it to be accepted.

Reviewer #3

(Remarks to the Author)
I confirmed. Now I recommend accept as it is.

Open Access This Peer Review File is licensed under a Creative Commons Attribution 4.0 International License, which permits use, sharing, adaptation, distribution and reproduction in any medium or format, as long as you give appropriate credit to the original author(s) and the source, provide a link to the Creative Commons license, and indicate if changes were made.
In cases where reviewers are anonymous, credit should be given to 'Anonymous Referee' and the source.
The images or other third party material in this Peer Review File are included in the article's Creative Commons license, unless indicated otherwise in a credit line to the material. If material is not included in the article's Creative Commons license and your intended use is not permitted by statutory regulation or exceeds the permitted use, you will need to obtain permission directly from the copyright holder.
To view a copy of this license, visit https://creativecommons.org/licenses/by/4.0/

Rebuttal Letter

Manuscript Title: Toward Smart Wearable Systems: Evolution from Stretchable Materials and Fibres to Intelligent Wearable Systems

Manuscript ID: COMMSMAT-26-0161-T

We sincerely thank the Editor and the Reviewers for their careful evaluation of our manuscript and for their constructive comments and suggestions.

We have undertaken a thorough revision of the manuscript, resulting in substantial changes to its structure, content, and critical analysis. Several new sections have been added, existing sections have been extensively revised and reorganized, new tables and figures have been incorporated, and the whole manuscript has been changed to provide a more critical and forward-looking perspective. We believe that these revisions have considerably strengthened the manuscript and addressed all concerns raised during the review process.

For the convenience of the Editor and Reviewers, substantially revised text is shown in blue in this marked-up version of the manuscript. Below, we provide a detailed point-by-point response to all comments.

Reviewer #1

"This manuscript presents a review of recent advances in wearable electronics, covering material innovations, mechanical design strategies, textile integration, wireless communication, and AI-enabled signal processing. The topic is timely and of interest, particularly given the increasing demand for soft, conformable, and garment-integrated sensing systems in healthcare and human–machine interfaces. Some comments are as follows:"

We thank the reviewer for the comments and appreciate the recognition that covers recent advances in electronics, materials, mechanical designs, textile integration, wireless communication, and artificial intelligence. We are pleased that the reviewer finds the topic timely and of interest. The authors thank the reviewer for constructive comments and insightful suggestions. We carefully considered each point and provided a detailed point-by-point response, together with the corresponding changes implemented in the revised manuscript.

Comment 1

"The title emphasizes: "From Rigid to Flexible, Stretchable Fibers", which suggests that the manuscript will focus on fiber-based electronics and textile-integrated sensing systems as the central theme. However, in its current form, only a relatively small portion of the manuscript (primarily Figure 3 and related discussion) is dedicated to fiber/yarn-based systems."

Response: We thank the reviewer for the comment. We agree that the original title may have suggested a stronger (or only) emphasis on fiber- and yarn-based electronics than was reflected in the manuscript.

Changes in manuscript: The manuscript title was revised from "The Future of Wearables: From Rigid to Flexible, Stretchable Fibers" to "Toward Smart Wearable Systems: Evolution from Stretchable Materials and Fibers to Intelligent Wearable Systems" to better match the comprehensive scope of the Review.

Comment 2

"While the manuscript provides a wide coverage of the field, it currently reads largely as a descriptive compilation of literature rather than a critical and forward-looking review. More importantly, there is a significant mismatch between the stated focus on fiber-based wearable systems and the actual content of the manuscript."

Response: We thank the reviewer for this insightful comment. We agree that the initial version of the manuscript placed greater emphasis on summarizing existing studies and positioning it in a more critical and looking forward will highly strengthen the review.

Changes in manuscript: Accordingly, the following revisions have been implemented:

- Added some critical evaluations and critical discussions throughout the manuscript, highlighting limitations and practical challenges, are marked in blue, in the track changes manuscript for clarity (e.g., issues that may arise from long-term wear, sweat, washing, manufacturability consistency, real-world calibration, fabrication costs, scalability concerns, reproducibility, if there is a requirements for complex micromachining or post-processing).

- Included comparisons Tables, for each device and highlighting the sensitivity/stretchability and fabrication method and demonstrated application.
- Moreover, to improve the comparative assessment of the different sensing strategies, we added a new radar-chart-based figure (Figure 6). The figure summarizes the clearly shows the strengths and limitations of composites, crack-engineered, auxetic, and fiber/yarn-based stretchable strain sensors in terms of sensitivity, stretchability, durability, textile compatibility, and ease of fabrication.
- Strengthened the discussion of emerging trends and future research directions. By adding a section titled "Challenges and Future Perspectives" to emphasize emerging research directions and unresolved issues.
- Revised the "Outlook" section to provide a more forward-looking perspective on opportunities and challenges for future development.

Comment 3

"The manuscript is organized into sections: materials, circuits, wireless, AI, but it lacks a coherent conceptual framework, which is not shown in Figure 1."

Response: We thank the reviewer for this comment.
Changes in manuscript: To address that, we include a new Figure 1 to clearly present the different topics covered.

Comment 4

"A major limitation of the manuscript is the absence of systematic quantitative benchmarking. Although individual values are occasionally mentioned (e.g., *GF*, strain limits)."

Response: We thank the reviewer for this important comment. We agree that the initial version of the manuscript lacked sufficiently systematic quantitative benchmarking across the reported studies.

Changes in manuscript: In response, we incorporated more comparative discussion throughout the text. Moreover, Tables 1-2 position the various approaches relative to one another with respect to performance metrics.

Comment 5

"Fiber electronics section lacks depth and novelty. For example: important missing topics such as thermally drawn multifunctional fibers."

Response: We thank the reviewer for this valuable comment. We agree that the original fiber electronics section could be strengthened further in depth and coverage.

Changes in manuscript: In response, we substantially revised manuscript we have a newly addded section about "Scalable Manufacturing and Fabrication Techniques" including additional emerging strategies, such as Thermally Induced technique. In particular, we added a section on thermally drawn multifunctional fibers, appears at pages 14-16.

Comment 6

"The manuscript mentions healthcare applications and digital outcome measures. However, there is limited discussion on real-world deployment such clinical validation, calibration and reproducibility, etc."

Response: We thank the reviewer for this important comment. We agree that the initial version of the manuscript provided limited discussion regarding the real-world translation of wearable electronic systems in real-world deployment.

Changes in manuscript: In response, we expanded the manuscript to address practical challenges in clinical implementation, such as reproducibility, long-term operational stability, and inter-user variability. These topics are now discussed in greater detail in the new "Challenges and Future Perspectives" section, which outlines current limitations and future requirements for adopting wearable and textile-integrated electronic systems.

These revisions have been incorporated throughout the manuscript, particularly in pages 26-27, and are in blue in the revised version.

Reviewer #2

"This paper provides a comprehensive review on the development of wearable electronics, focusing on the technological transition from rigid devices to flexible, stretchable fiber integrated systems. It systematically summarizes advanced materials, mechanical design strategies, stretchable conductive circuits, textile based wireless communication, multimodal signal fusion, and AI integration for digital health applications. The review also discusses current challenges including system integration, data security, privacy issues, and ethical concerns, as well as future directions for soft wearable technologies. The topic is highly relevant to flexible electronics and smart textiles, and the review covers sufficient scope with clear logic. However, several improvements in presentation, structure, readability, and technical description are needed to enhance its quality and academic impact."

We sincerely thank the reviewer for evaluating our manuscript and recognizing the relevance and timeliness of the topic. We appreciate the assessment that the manuscript provides a comprehensive and logically organized overview of the evolution of wearable electronics, covering advanced materials, mechanical design strategies, stretchable conductive circuits, textile-based wireless communication, multimodal signal fusion, AI integration, and associated challenges and future directions. We have refined the organization of several sections, improved the flow between topics, clarified technical descriptions where necessary, and enhanced the presentation of figures and tables. Detailed responses to each specific comment are provided below.

Comment 1

"Figures throughout the manuscript vary significantly in size, layout, and annotation style. All figures are too small to clearly show structural details, working principles, and fabrication flows."

Response: We thank the reviewer for this valuable comment. We agree that the original figures lacked sufficient consistency in layout, annotation style, and visual clarity.

Changes in manuscript: In response, we revised all figures in the manuscript to improve readability, consistency, and presentation quality.

Comment 2

"The scale, font, and labeling of subfigures are inconsistent, which greatly reduces readability and visual coherence. All figures should be standardized, enlarged, and uniformly formatted."

Response: We thank the reviewer for this important observation.

Changes in manuscript: In response, all figures and subfigures throughout the manuscript were carefully revised and reformatted to ensure a more uniform presentation.

Comment 3

"The section "Material & Mechanical Innovations (From Rigid Systems to Stretchable Textiles via Fiber-Level Integration)" covers a wide range of content including conductive materials, percolation theory, crack engineering, auxetic structures, breathability, fiber fabrication, and textile integration. All these topics are merged without clear subsections, making the logic difficult to follow. It is strongly suggested to divide this section into multiple clear subheadings. Other sections should also consider this division optimization."

Response: We thank the reviewer for this constructive suggestion.

Changes in manuscript: In response, we reorganized this section into multiple clearly defined subsections to improve readability and logical flow.

Specifically, separate subheadings were introduced within all manuscripts, as follows:

- Highly Stretchable Composites
  - Crack Engineering
  - Auxetic Architectures
- Fiber- and Yarn-Based Strain Sensors
  - Coated Conductive Fibers
  - Structural Fiber Strategies (helical/coiled, twisted, braided, interlaced)
  - Structural Fiber Architectures (helical/coiled, twisted, braided, interlaced)
- Scalable Manufacturing and Fabrication Techniques

- Quantitative and Qualitative Assessment of Reported Wearable Sensors
- Structural Engineering Strategies for Stretchable Circuits and Interconnects
  - Structural Engineering Approaches
    - Buckling/Wrinkling Engineering
    - Serpentine Interconnect Engineering
    - Kirigami/Origami Engineering
  - Material-Based Approaches
    - Conductive Polymer Composites and Intrinsically Stretchable Conductive Polymers
    - Hybrid Soft–Rigid Interconnection Engineering
    - Liquid Metal-Based Interconnections
- Wireless Communication Through Textile Solutions
- Signal Processing, Multi-modal Fusion, and AI Integration
  - Signal Processing
  - Multi-modal Fusion
  - AI Integration
- Challenges and Future Perspectives
- Outlook

Comment 4

"Many paragraphs switch abruptly between materials, mechanisms, and applications without smooth connecting statements. Transitional sentences and clear topic guidance are needed."

Response: We thank the reviewer for this valuable observation.

Changes in manuscript: In response, we revised the manuscript to improve the logical flow by adding clear transitions, topic introductions, and concluding statements in the major sections. We also reorganized paragraphs to better connect material properties, device mechanisms, and related applications within a coherent framework. Some examples are as follows: "In general, crack engineering has become an effective approach...fabrication complexity and limits process reproducibility", "Another aspect that researchers should consider...subjected to tensile strain due to the Poisson effect", "Despite advancements in sensitivity/stretchability, film-based strain sensors face important limitations...critical challenge for the widespread adoption of thin-film-based wearable sensors", "Fiber-based electronic devices (FEDs) have emerged...offer mechanical flexibility and easy integration into wearable garments", "Beyond coating, metal-nanowire composites can be used as scaffolds on stretchable fiber for high-performance mechanical sensing", "In addition to surface engineering methods such as prestretching and crack or wrinkle formation... sensing performance of fiber-based strain sensors", "Despite the remarkable sensing performance reported for many laboratory-scale strain sensor...fiber-based strain sensors that can be easily translated to industrial production", marked up in blue.

Comment 5

"The opening statement covers an overly broad range of wearable devices, while the manuscript mainly focuses on stretchable fiber-based composite devices and textile-integrated systems. The core theme, scope, and logical outline of the review are not clearly highlighted. The authors should rewrite and narrow the introduction to clearly state that this review concentrates on fiber-based stretchable wearable electronics, rather than general wearables."

Response: We thank the reviewer for this insightful comment. We agree that the original introduction was not sufficiently emphasize the focus and scope of the review. While we agree that a clear and focused introduction is essential, the objective of this review is to provide a system-level perspective on wearable electronic platforms rather than focusing only to a subtopic such as fiber-based stretchable wearable electronics.

Changes in manuscript: To address this, we revised the Introduction to better reflect the review's broader scope.

Comment 6

"Stretchable devices and stretchable circuits have inherent overlaps in their preparation methods. This article specifically discusses the content of stretchable circuits, but it has little relevance to the previously mentioned fiber-based stretchable devices for fabrics. Careful consideration is needed for classification, and it is necessary to optimize it carefully to ensure a more logical structure. The current structure is rather chaotic."

Response:

We thank the reviewer for this insightful comment. We agree that clearer transitions and topic guidance are important for improving readability. While similar structural design concepts (e.g., pre-strain engineering, wrinkle engineering etc) are employed in both domains, their objectives differ: sensors are designed to optimize signal transduction and sensing performance, whereas circuits and interconnects are intended to ensure reliable power delivery, signal transmission, and electrical stability under mechanical deformation.

Changes in manuscript: In response, we have revised the manuscript structure and added transitional statements to better highlight the relationship between the different sections To further improve clarity, we have added a new comparison table (Table 3) summarizing the key distinctions between wearable sensors and stretchable circuits/interconnects in terms of their functions, design objectives, and performance requirements.

Reviewer #3 (Remarks to the Author): Reviewer Comments — Recommendation: Major Revision (borderline reject)

"The manuscript surveys the transition from rigid to fiber-based stretchable wearables, and the authors have clearly invested effort in the figures (Figure 2 on percolation/cracking/auxetic mechanics and Figure 3 on fiber manufacturing are pedagogically well-drawn) and in the regulatory section, which is a relatively uncommon addition to a hardware-focused review. With 356 references, the literature base is also broad. In its current form, however, the review does not establish a distinct contribution alongside an unusually crowded recent literature, and the scope mismatch with the title needs to be addressed before the manuscript can be evaluated on scientific merit."

Response: We sincerely thank the reviewer for the thoughtful and constructive assessment of our manuscript. We appreciate the reviewer's positive comments regarding the pedagogical quality of the figures, the breadth of the literature coverage, and the inclusion of the regulatory and ethics perspective, we agree that the unique contribution of the review should be clearly articulated.

Changes in manuscript: For the two concerns that the reviewer raised. 1. Scope mismatch with the title 2. Lack of a clearly articulated unique contribution compared to existing literature, we did the following:

To address the title–scope mismatch, we revised both the title and the Introduction to better reflect the review's broader scope. Specifically, we have revised the Introduction to more explicitly define the manuscript's scope and distinguishing features. Therefore, the unique contributions of this Review paper are that it provides:

- A critical evaluation of the trade-offs between sensing performance and practical implementation requirements, including sensitivity, stretchability, manufacturability, and textile integration, which are essential for the development of reliable and commercially viable wearable electronic systems.
- A dedicated discussion of scalability and manufacturability challenges, aspects that have received comparatively limited attention in many existing review articles.
- Consideration of ethical, privacy, and data-security challenges, which represent critical barriers to the responsible large-scale deployment of wearable technologies.

We believe that this integrated perspective distinguishes the present work from many recent reviews that focus on individual device components or specific application areas, as discussed now in the Introduction.

"The most fundamental concern is that the differentiator is not made. The last 24 months have produced multiple specialist reviews that cover each of the manuscript's sub-sections in substantially greater depth, none of which are positioned against in the introduction: Yin, Wang, Tat and Chen in Nat Rev Bioeng (2024) on motion artefact management for soft bioelectronics, integrating materials, device design, and algorithmic intervention; Xu, Xiao and Chen in Natl Sci Rev (2024) on stretchable fiber strain sensors for biomonitoring; Liu et al. in Nano-Micro Lett (2022) on functionalized fiber-based strain sensors; Chen, He, Du and Lee in Nano Energy (2023) on textile-based wearable electronics through the fiber–yarn–fabric hierarchy; Xu et al. in Nano-Micro Lett (2025) on smart textiles for personalized sports and healthcare; the recent system-on-fiber review in Adv Sci (2025); and Fang et al. in Adv Mater (2025) on ionic conductive textiles. The introduction must explicitly state what gap this review fills relative to those works. As written, an informed reader cannot answer "why this review, why now.""

Response: The reviewer raises an important point, and we agree that the original Introduction did not sufficiently articulate this review's unique positioning relative to several recent high-quality specialist reviews. In the revised manuscript, we will substantially strengthen the Introduction to explicitly define the gap our review addresses and position it within the existing literature.

Changes in the manuscript: To address this concern, in the revised manuscript, a dedicated paragraph has been added comparing our scope with recent key reviews (including the works suggested by the reviewer). The revised text is provided in the Introduction (Pages 1-2), marked in blue. We also define the unresolved gap motivating this review, clarify the rationale for "why now", particularly in the limitations imposed by current technological and healthcare infrastructures, as well as ethical considerations, which are often discussed only as secondary considerations despite their importance for real-world implementation. Sections: "Although numerous specialized reviews... wireless communication, signal processing, and system-level integration remain highly valuable.".

"The title–scope mismatch is the second concern and is, in my view, the issue most likely to determine the manuscript's fate at Communications Materials. The title commits the review to fibers, but only roughly a third of the body actually concerns fiber-level engineering; the remainder spans thin-film stretchable conductors, stretchable circuits, wireless communication, signal processing/AI, and ethics. Each of these is a legitimate sub-field, but each is treated at tutorial depth, and as a result no

single sub-section reaches the specialist-level contribution that Communications Materials readers expect. The authors should either (i) honestly narrow the scope and the title to fiber-level innovations and engage with the specialist literature above in real depth, or (ii) keep the broad scope but explicitly reposition the review around an integrating thesis that the existing surveys do not provide — for example, the systems-engineering challenges of going from a fiber to a garment-level closed loop. The current uncommitted middle position is the weakest of the three options."

Response: We thank the reviewer for this insightful assessment and agree that the previous version occupied an insufficiently refined middle ground between a specialist fiber-focused review and a broader systems-level perspective article. In response, we have substantially revised the manuscript to adopt the latter positioning explicitly and consistently throughout the title, Introduction, section organization, and concluding outlook.

Changes in manuscript: The manuscript is structured to guide the reader through the key technological layers required for wearable electronic systems, including (i) functional materials, (ii) thin-films (iii) structural engineering strategies, (iv) fiber-, and textile-based sensing platforms, (v) scalable and manufacturable wearable systems (vi) stretchable circuits and interconnects, wireless communication technologies, (vii) AI-assisted data processing, and (vii) Challenges (including considerations for close loop system, system reliability, data security, and ethical considerations).

We believe that this systems-oriented and cross-disciplinary perspective differentiates the present review from many existing surveys that focus primarily on individual materials, sensing mechanisms, or device categories. To further clarify this positioning, we have revised the Introduction and added text highlighting the relationships between the different technological layers discussed throughout the manuscript.

"Technical depth and quantitative anchoring are also insufficient for a 2026 review on this topic. The materials section repeatedly cites gauge factors and stretchability values from individual studies without consolidating them into a comparison table, so a reader cannot easily place a new device against the field. The stretchable-circuits section invokes the standard buckling/stiffness/intrinsic taxonomy without adding new analytical structure on top of it. At least one summary table consolidating gauge factor, stretchability range, response time, washing-cycle stability, and integration method across the major fiber sensor families would substantially strengthen the review.

Response: We thank the reviewer for this thoughtful and constructive assessment. We agree that the original manuscript emphasized descriptive coverage more strongly than quantitative synthesis in several sections. In response, we have substantially revised the review to improve technical consolidation, benchmarking, and analytical depth.

Changes in manuscript:

To improve the technical depth and quantitative grounding of the review, we added summary tables (Tables 1-2). We also conducted both quantitative and qualitative analyses of the literature reviewed in the manuscript, extracting key performance metrics reported in the original studies. Three quantitative metrics are maximum sensitivity (GF), Maximum working range (stretchability), and durability, as shown in Supplementary Table S1. In addition, we introduced two qualitative metrics: Textile compatibility and Ease of fabrication. These parameters were assessed based on criteria such as whether the sensing system was directly integrated into garments and the extent of additional fabrication or post-processing steps required. Each study was individually evaluated, and a detailed explanation of the scoring methodology is provided (Supplementary Table S2). The assigned scores were averaged to facilitate a comparative assessment across the reviewed studies. We should note that certain parameters suggested by the reviewer, such as response time (for example, response time is predominantly emphasized in pressure-sensing studies rather than strain-focused fiber systems) and washing-cycle durability, is not consistently reported across the literature and are often application-specific. However, within the manuscript we make critical discussions about that aspects, where relevant.

For the stretchable-circuits section to improve the analytical depth of the section, we have revised the section "Structural Engineering Strategies for Stretchable Circuits and Interconnects" to more explicitly and we summarized the studies by adding a new Table (Table 5) to facilitate cross-strategy evaluation.

"The wireless and AI/signal-processing sections describe CNN, RNN, multimodal fusion, and edge intelligence at a level that does not engage with current state-space, model-architecture, or on-device-inference details. Two further points deserve mention. The author team's real strengths — wearable signal processing and digital biomarkers in the BMHT group at ETH, and physiological-signal AI from the Khalifa side — do not come through clearly in the manuscript, because the AI/signal-processing section reads as a generic survey rather than as an authored perspective. I would strongly encourage the authors to use the revision to bring their own programmatic viewpoint to the front, particularly in the signal-processing and digital-biomarker discussions; this would also be the most natural way to manufacture a real differentiator.

Response: We thank the reviewer for this thoughtful suggestion. We agree that an author-specific perspective can add value in focused review or perspective articles. However, the objective of the present manuscript is to provide a broad and

balanced overview of recent advances in wearable technologies, with particular emphasis on materials, device architectures, and system-level developments. We hope this rationale clarifies the intended focus of the manuscript.

"The manuscript also needs sentence-level English editing throughout: there are recurrent issues such as "rigid electronics may delaminate due to displacement," "smart appear" (apparel), "battery offerings," "harware-level paradigm shoft," and several broken constructions. For a senior-authored review this level of polish is below what reviewers will expect."

Response: We thank the reviewer for these valuable observations.

Changes in manuscript:
We have also completed comprehensive language editing to improve clarity, grammar, terminology, and sentence structure throughout. All examples highlighted by the reviewer, as well as similar issues, have been addressed to ensure the manuscript meets the expected standard of English.

"Finally, the rpleaseegulatory/ethics section, while uncommon for a hardware review and a potentially valuable differentiator, is currently treated at the same depth as the rest of the manuscript and reads more as a checklist than as an analysis. If the authors choose to keep this section, it would benefit from being explicitly framed as one of the review's distinguishing contributions, with concrete engagement with how GDPR, the EU AI Act, and SaMD pathways change design decisions for fiber-based wearables specifically."

Response: We thank the reviewer for this insightful comment. While the primary focus of this review remains the materials, device architectures, and fiber-based wearables, we expanded the regulatory and ethics subsection to provide a more analytical discussion of how GDPR, the EU AI Act, and Software-as-Medical-Device frameworks influence design, validation, data management, and translation pathways for wearable systems. We want to highlight that this discussion is intended as an important translational consideration for the field rather than serve as a comprehensive regulatory review.

Changes in manuscript: In response, we have revised this section to more explicitly discuss how regulatory requirements, including GDPR, the EU AI Act, and Software as a Medical Device (SaMD) pathways, influence the design, deployment, and validation of wearable electronic systems. In particular, we highlight how considerations related to data governance, privacy protection, AI transparency, cybersecurity, and clinical validation may affect system architecture, data-processing strategies, and the translation of wearable technologies from laboratory demonstrations to real-world healthcare applications.

We have also strengthened the Introduction and concluding sections to emphasize that the discussion of regulatory and ethical challenges constitutes an additional translational perspective provided by this review.

"In summary, without (i) an explicit gap statement against the 2024–2026 reviews above, (ii) resolution of the title–scope mismatch through either narrowing or repositioning, (iii) real technical depth and quantitative anchoring in at least the materials and circuits sections, and (iv) sentence-level editing throughout, the manuscript overlaps too heavily with stronger recent reviews to justify publication in Communications Materials. With these revisions, however, the work could become a useful entry point to a difficult literature, particularly if the authors lean into their signal-processing and regulatory perspectives as the differentiating axes. I look forward to a revised version."

Response: We thank the reviewer for this constructive and encouraging assessment.

Changes in manuscript:
In response, we:

- Clarified the unique contribution of the review by emphasizing the need for a comprehensive perspective in this rapidly evolving field. The revised manuscript now more clearly highlights the connections between functional materials, structural engineering strategies, sensing technologies, stretchable circuits and interconnects, wireless communication, artificial intelligence, ethical considerations, privacy and data-security challenges.
- Revised the title and Introduction to better align with the broad scope and system-level perspective of the review, thereby more accurately reflecting the range of topics covered throughout the manuscript.
- Strengthened the discussion of the trade-offs between sensing performance and practical implementation requirements, including stretchability, manufacturability, scalability, durability, and textile integration.
- Carefully revised the manuscript for language, clarity, and readability, and checked the English throughout the text.

We believe that these revisions substantially improve the manuscript and more clearly establish its positioning and contribution within the existing review literature.
